# Parenting Training Plus Behavioral Treatment for Children With Obesity: A Randomized Clinical Trial

**DOI:** 10.1001/jamanetworkopen.2025.8398

**Published:** 2025-05-05

**Authors:** Kyung E. Rhee, Takisha Corbett, Shamin Patel, Dawn M. Eichen, David R. Strong, Eastern Kang-Sim, Cheryl A.M. Anderson, Bess H. Marcus, Kerri N. Boutelle

**Affiliations:** 1 Department of Pediatrics, University of California, San Diego School of Medicine, La Jolla; 2 Herbert Wertheim School of Public Health and Human Longevity Science, University of California, San Diego, La Jolla; 3 Department of Behavioral and Social Sciences, School of Public Health, Brown University, Providence, Rhode Island; 4 Department of Psychiatry, University of California, San Diego School of Medicine, La Jolla

## Abstract

**Importance:**

Family-based behavioral treatment (FBT) is recommended for childhood obesity treatment; however, it is not effective for all families. Since parenting training (PT) has been associated with healthy weight and eating behaviors, intensive PT may augment delivery of behavior change strategies and improve child weight loss outcomes.

**Objective:**

To compare the efficacy of child overweight or obesity treatment that adds intensive PT to standard FBT with the efficacy of FBT alone.

**Design, Setting, and Participants:**

This 2-arm randomized clinical trial (Reinforced, Enhanced, Families, Responsibility, Education, Support, and Health [ReFRESH]) conducted from April 2017 to November 2022 at an academic center in San Diego, California, included children aged 7 to 12 years with overweight or obesity (body mass index [BMI]≥85th to <99.9th percentile) and one of their parents.

**Interventions:**

Parent-child dyads were randomized 1:1 to the intervention group, which received FBT plus PT, or the control group, which received FBT alone. Both groups received twenty 60-minute sessions over 6 months with separate parent and child groups led by staff and nine 20-minute behavior change coaching sessions. The FBT plus PT group sessions incorporated additional intensive parenting skills training in an interactive format.

**Main Outcomes and Measures:**

The primary outcome was change from baseline in child BMI *z* score and BMI as a percentage of the 95th BMI percentile (BMIp95) after treatment (month 6) and at 6- and 12-month follow-up. Secondary outcomes included the proportion of children who attained clinically meaningful weight loss (ie, reduction of ≥0.20 BMI *z* score units) and intervention dropout rates. Intention-to-treat analysis was conducted using linear mixed models and logistic regression.

**Results:**

A total of 140 parent-child dyads were included, with 70 in each treatment arm. Mean (SD) child age was 9.91 (1.54) years, and baseline BMI *z* score was 2.28 (0.80); 71 children (50.7%) were female. There were no significant between-group differences in BMI *z* score or BMIp95 after treatment or at the follow-up time points. Both groups had significant decreases in weight status after treatment (combined BMI *z* score: β, −0.14 [95% CI, −0.21 to −0.07]; *P* < .001; combined BMIp95: β, −3.46 [95% CI, −5.41 to −1.51]; *P* < .001). More children in the FBT plus PT arm compared with the FBT arm had a reduction of at least 0.20 BMI *z* score units (34 [48.6%] vs 22 [31.4%]; *P* = .01) after treatment (adjusted odds ratio, 2.10 [95% CI, 1.01-4.47]). Both treatments were well accepted, with no between-group differences in risk of dropout (hazard ratio, 1.01 [95% CI, 0.72-1.43]).

**Conclusions and Relevance:**

In this randomized clinical trial examining the effect of parenting training on child weight status, there were no significant differences in weight status between groups; children in both groups had a significant reduction in weight status. However, more children had clinically meaningful weight loss in the FBT plus PT group. Further work is needed to determine factors associated with treatment response and changes in parenting skills.

**Trial Registration:**

ClinicalTrials.gov Identifier: NCT02976636

## Key Points

**Question:**

Does the addition of more intensive parenting training (PT) to traditional family-based behavioral treatment (FBT) for childhood overweight or obesity improve weight loss outcomes?

**Findings:**

In this randomized clinical trial of 140 parent-child dyads, children in both the FBT plus PT and FBT only groups had a significant decrease in weight status, but there was no difference between groups.

**Meaning:**

The findings suggest that FBT reduces weight status in children with overweight or obesity but that additional intensive PT does not further improve outcomes.

## Teaser

This randomized clinical trial compares the effectiveness of behavior change strategies using family-based behavioral treatment (FBT) plus parenting training vs FBT alone to improve weight loss outcomes in children with overweight or obesity.

## Introduction

Intensive health behavior and lifestyle treatment (IHBLT) for childhood obesity is one of the most effective treatments for pediatric weight management and is recommended by the American Academy of Pediatrics^1^ and US Preventive Services Task Force^2^ for children aged 6 years or older with overweight or obesity. However, not all children respond to IHBLT programs, with some studies suggesting that only 30% to 40% of children show any decrease in body mass index (BMI) percentile (due to weight loss or weight maintenance with continued growth) and that a similar proportion maintain their BMI percentile.^3,4,5,6^ While weight loss medication may be an option for some teenagers (and should be combined with IHBLT), the long-term effects of these medications are unknown^7^ and they are not yet an option for younger children. Therefore, additional behavioral treatment options are still needed to improve outcomes.

One such option may be more intensive parenting training (PT). Currently, family-based behavioral treatment (FBT), one form of IHBLT, teaches parents behavioral strategies needed to change and reinforce healthy diet and physical activity behaviors in children. Parenting training may be a useful addition because it provides focused training on how to deliver and implement these behavioral strategies. Parenting training promotes the use of an authoritative parenting style, which balances the use of firm rules, structure, and boundaries in a warm and supportive manner.^8^ Since parenting styles are thought to provide the context in which specific behavioral strategies are delivered and interpreted by the child,^8^ a program that incorporates more targeted parenting skills training may be able to modify or strengthen the impact of the behavioral strategies that are taught in FBT.^9,10,11,12^

Additionally, an authoritative parenting style has been associated with lower BMI and normal weight status^13,14,15,16^ as well as healthier eating behaviors.^17,18,19,20,21^ Given these findings, 2 groups in Australia tested the effect of PT on pediatric weight loss for school-age children.^22,23^ These researchers added lifestyle training to a generalized parenting program for obesity management and compared it with either a waiting list control^22^ or the parenting program alone.^23^ These studies found that PT combined with a lifestyle intervention led to a decrease in BMI *z* scores (mean change, −0.20 units) after treatment and at 1-year follow-up.^22,23^ These changes were obtained with a 12-week program involving nine 90-minute group sessions and three 20-minute phone sessions. While these programs obtained changes in BMI *z* score comparable to those with traditional FBT, the studies did not compare this program with FBT alone.

The goal of the Reinforced, Enhanced, Families, Responsibility, Education, Support, and Health (ReFRESH) study was to adapt the current FBT program to include a greater focus on parenting skills training and compare the efficacy of this new program (FBT plus PT) with standard treatment (FBT) alone among children aged 7 to 12 years with overweight or obesity and one of their parents. We chose to focus on this age group since parents are more involved in their child’s lifestyle behaviors during this time and parenting during adolescence focuses on different skills. We hypothesized that FBT plus PT would show greater decreases in child weight status compared with FBT after treatment and at 6- and 12-month follow-up. We also examined the proportion of children who had clinically meaningful weight loss (decrease of ≥0.20 BMI *z* score units).^24^ Our secondary aims were to examine rates of dropout, treatment adherence, and acceptability in both groups given the increased intensity of parenting skills training and change in treatment format in FBT plus PT. Our exploratory aim was to examine factors associated with greater weight loss.

## Methods

### Study Design

This 2-arm, parallel assignment randomized clinical trial (NCT02976636) was conducted from April 2017 to November 2022. The trial was approved by the University of California (UC), San Diego Human Research Protections Program (trial protocol available in Supplement 1). A participating parent provided written consent, and the child provided written assent. Due to the COVID-19 pandemic and challenges with recruitment, the original sample was reduced from 160 to 140 parent-child dyads. The study followed the Consolidated Standards of Reporting Trials (CONSORT) reporting guideline.

### Setting

The study was conducted at the UC San Diego Center for Healthy Eating and Activity Research. Families participated at 1 of 2 office sites in La Jolla and San Marcos, California. Because of the COVID-19 pandemic, families were transitioned to virtual group sessions using an online videoconferencing platform with a Health Insurance Portability and Accountability Act–compliant, password-protected format in March 2020.

### Participants and Recruitment

Families were included in the study if the child was aged 7 to 12 years and had overweight (BMI ≥85th to <95th percentile) or obesity (BMI ≥95th to <99.9th percentile) (the BMI categories were chosen to decrease risk of developing comorbidities and need for medical intervention),^25^ parents were willing to attend 20 weekly group sessions and complete assessments, and parents spoke English at a fifth-grade level or higher. Families were excluded if the child was taking medication that affected their appetite or weight, the child had severe developmental delay or disability that would affect participation, the child or parent had a psychiatric illness that would limit treatment participation (eg, eating disorder, conduct disorder, psychosis, or suicidality), and/or the family was planning to move out of the area within the time frame of the study.

Families were recruited through pediatric primary care networks in San Diego County, online advertisements, community events, and ResearchMatch.^26^ Families who potentially met inclusion criteria were also identified in the electronic health record and were sent electronic messages or postal letters to inform them of the research opportunity. Interested parents completed an online screening tool and phone screening and were invited to an orientation meeting if they met initial eligibility criteria.

### Randomization and Intervention

Families were randomized 1:1 using block design (blockrand package in R, version 1.5 [R Project for Statistical Computing]^27^) based on child sex and parent weight (healthy weight vs overweight or obesity) by the study statistician (D.R.S.), who was blinded to study participants. Treatment occurred over 6 months with a 12-month follow-up period; details regarding the intervention and protocol have been published in detail elsewhere.^28^ In brief, both arms of the study included traditional FBT that addressed nutrition, physical activity, behavior change strategies, and basic parenting skills. The intervention arm (FBT plus PT) condensed the FBT training and included additional intensive parenting skills training using a more interactive training format (eg, role plays). Both arms participated in 60-minute group sessions weekly for 16 weeks, then every other week for 8 weeks (totaling 20 sessions over 6 months). Parents and children in both arms also received 20-minute behavior change coaching sessions every other week (totaling 9 sessions). In these sessions, the behavior coach reviewed self-monitoring records and problem solved any barriers to program implementation. Parents and children were weighed weekly, and the goal in both arms was to decrease weight by 0.23 to 0.45 kg/week.^29^ The same number of staff were used in each study arm; training and supervision for staff were differentiated by study group. Ten percent of each treatment group’s recordings and behavioral coaching sessions were reviewed to ensure treatment fidelity. The first 3 cohorts received all of their treatment in person. Cohorts 5 and 6 received all of their treatment remotely. Cohort 4 received the first 16 sessions in person and was then switched to remote for the last 4 sessions. Assessments occurred at baseline, during treatment, after treatment (month 6), at 6-month follow-up (month 12), and at 12-month follow-up (month 18) (a timeline is given in eTable 1 in Supplement 2).

### FBT

FBT provides the following recommendations: decreasing food portion sizes and foods that are high in fat and added sugar and increasing healthy food options, such as fruits and vegetables, according to the US Department of Agriculture MyPlate guidelines^30^; increasing physical activity to at least 90 minutes per day for 5 of 7 days; and decreasing sedentary activities (eg, screen time). FBT also promotes behavioral strategies for weight loss, such as self-monitoring, stimulus control, problem solving, goal setting, planning ahead, cognitive restructuring, and basic parenting strategies, such as positive reinforcement, modeling, and use of reward systems. The content for child groups was modified to be age appropriate, and parenting strategies were not discussed.

### FBT Plus PT

The FBT plus PT group included all components of FBT plus more intensive parenting education that was reinforced using active skills training. The active parenting skills training was integrated into each session and focused on how to use appropriate parenting techniques to create a positive setting in which to implement the behavior changes taught in FBT (eTable 2 in Supplement 2 has detailed treatment content). Active skills training included video modeling with discussion, role play with feedback, and behavioral practice during group sessions. These methods have been shown to be effective at producing behavioral changes^31,32^ and are not currently used in traditional FBT. Parenting components were adapted from the Incredible Years program^33^ and parent-child interaction therapy^34^ and focused on positive parenting fundamentals.^35,36^ Parenting topics included in FBT plus PT that are not currently included in FBT were validating children’s emotions, helping children persist with healthy choices, using more effective discipline strategies (eg, direct commands and forced choice) and limit-setting skills, setting structure and consequences, and regulating emotions.

### Main Measures and Outcomes

The primary outcome was change in child weight status measured as BMI *z* score.^37^ We also included change in BMI as a percentage of the 95th BMI percentile (BMIp95), which is recommended for analysis in longitudinal weight management trials and for children with very high BMI (>97th percentile).^38,39^ Clinically meaningful weight loss was defined as a decrease in BMI *z* score of at least 0.20 units.^24^ Anthropometric data were obtained using a Seca 222 mechanical telescopic measuring rod (Seca GmbH) and a Tanita digital scale (model WB-110A; Tanita Corp of America). Height was recorded to the nearest 0.1 cm, and weight was recorded to the nearest 0.1 kg; the mean of 2 values was used for analysis. During the pandemic, Bluetooth-enabled scales (Withings) were sent to families to collect weights, and tape measures were used to collect height.

Parenting behaviors were measured using the Comprehensive General Parenting Questionnaire.^40^ This is an 85-item parent self-report questionnaire that measures 5 constructs of parenting: nurturance, behavioral control, structure, overprotection, and coercive control. Patient demographic data included child and parent age, sex, race and ethnicity (self-reported using 2017 National Institutes of Health–defined options), and parent educational level (high school diploma or less vs some college or more). Race and ethnicity were included in the analysis because we did not collect detailed measures of socioeconomic status and discrimination, and these constructs may reflect how people experience the world around them. Categories were African American or Black, Asian, Hispanic or Latino, White, multiracial or other (a separate option with no further detail provided by participants), and unknown.

Attendance at weekly treatment sessions was tracked by group leaders. Dropout rates and adverse events were monitored throughout the study. Parents and children were asked to complete a satisfaction survey at the end of the study to determine overall liking of the study and usefulness of the different strategies that were taught (using 5-point Likert scales, with 1 indicating “not at all useful” and 5, “very useful”).

### Blinding

Given the nature of the study, participants could not be blinded to group assignment. However, participants were blinded to the specific details of the research hypothesis. The study investigators (K.E.R., C.A.M.A., B.H.M., K.N.B.), assessor (D.M.E.), and statisticians (D.R.S., E.K.-S.) were blinded to the participant’s study group.

### Power and Sample Size

Empirical power and sample size calculations were conducted to support the primary outcome. Based on prior literature, we expected changes in BMI *z* score of −0.07 to −0.15 in the FBT group^41^ and changes of at least −0.11 to −0.30 in the FBT plus PT group.^22^ We generated 10 000 multivariate normal random samples of correlated outcomes using covariance estimates from previous trial data^3,16,22,41^ (range, 0.35-0.55) and analyzed the datasets using linear mixed-effects models for repeated assessments of BMI *z* score. The percentage of datasets with significant effects for greater reductions in BMI *z* score in the FBT plus PT group compared with the FBT group provided a simulation-based estimate of power. Assuming a combined sample size of 160 and setting α at 0.05, we had more than 81% power to detect treatment × time effects, allowing for up to 20% attrition. With the recruitment of 140 participants, new empirical estimates suggested power was reduced but remained in an acceptable range of more than 79% for the primary outcome.

### Statistical Analysis

Primary outcome evaluation used an intention-to-treat principle and linear mixed models^42,43,44^ implemented in R, version 4.4.^45^ Change in BMI *z* score and BMIp95 were used as primary outcomes with planned covariates of race and ethnicity and baseline weight status of the child and parent. Fixed effects included coefficients for rate of weight loss over time, treatment differences in weight, and treatment differences in the rate of weight loss over time. All *P* values were 2-sided with significance set at *P* < .05. Missing BMI *z* score and BMIp95 values were imputed using multilevel multiple imputation with all available time points.^46,47^ Logistic regression models were used to examine whether odds of attaining clinically meaningful weight loss differed by group and what factors were associated with attaining it.

Analyses of secondary outcomes examined rates of dropout, treatment adherence, and acceptability of FBT plus PT compared with FBT. We used survival analysis to assess the effect of FBT plus PT compared with FBT in decreasing risk for treatment dropout (defined as participant refusal to continue treatment or failing to return to treatment sessions without notification). Regression models for number of sessions attended quantified any differences in the probability of completing FBT plus PT vs FBT. Sex, age, and race and ethnicity were included to assess differential dropout between demographic groups. Parent ratings of treatment acceptability and whether they believed the components included in FBT plus PT or FBT addressed issues that were relevant to them were used to compare differences in acceptability.

## Results

**Figure 1.**
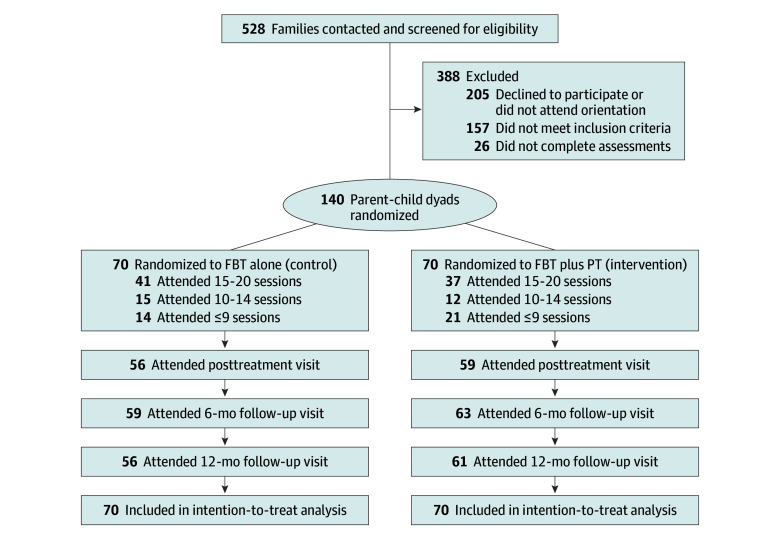
CONSORT Flow Diagram FBT indicates family-based behavioral treatment; PT, parenting training.

We enrolled 140 parent-child dyads, with 70 in each treatment arm (Figure 1). Children had a mean (SD) age of 9.91 (1.54) years and a mean (SD) BMI *z* score of 2.28 (0.80); 71 (50.7%) were female, and 69 (49.3%) were male. Five children had unknown race and ethnicity; of the remaining 135, 13 (9.6%) were African American or Black; 3 (2.2%), Asian; 74 (54.8%), Hispanic or Latino; 34 (25.2%), White; and 11 (8.1%), multiracial. Most parents were female (131 [93.6%]); 9 (6.4%) were male. Mean (SD) parent age was 41.3 (6.6) years. Demographics of the FBT plus PT and FBT groups are reported in Table 1.

**Table 1.** Participant Characteristics by Group

Characteristic	Participants^a^		
	Overall (N = 140)	FBT (n = 70)	FBT plus PT (n = 70)
**Parent**			
Age			
Mean (SD), y	41.3 (6.6)	41.1 (6.6)	41.6 (6.7)
Unknown, No.	1	0	1
Sex			
Female	131 (93.6)	67 (95.7)	64 (91.4)
Male	9 (6.4)	3 (4.3)	6 (8.6)
Race and ethnicity			
African American or Black	5 (3.6)	3 (4.3)	2 (2.9)
Asian	6 (4.4)	2 (2.9)	4 (5.9)
Hispanic or Latino	74 (54.0)	37 (53.6)	37 (54.4)
White	51 (37.2)	26 (37.7)	25 (36.7)
Multiracial or other^b^	1 (0.7)	1 (1.4)	0
Unknown	3	1	2
≤High school diploma	16 (11.4)	7 (10.0)	9 (12.9)
BMI, mean (SD)	39.81 (9.45)	39.50 (9.50)	40.12 (9.45)
Parenting style score, mean (SD)^c^			
Nurturance	4.44 (0.49)	4.37 (0.53)	4.52 (0.44)
Behavioral control	4.26 (0.34)	4.24 (0.37)	4.28 (0.32)
Structure	3.95 (0.38)	3.97 (0.37)	3.93 (0.39)
Overprotection	1.97 (0.55)	1.98 (0.59)	1.96 (0.50)
Coercive control	3.07 (0.52)	3.07 (0.52)	3.06 (0.53)
**Child**			
Age			
Mean (SD), y	9.91 (1.54)	9.86 (1.50)	9.97 (1.59)
Unknown, No.	1	0	1
Sex			
Female	71 (50.7)	35 (50.0)	36 (51.4)
Male	69 (49.3)	35 (50.0)	34 (48.6)
Race and ethnicity			
African American or Black	13 (9.6)	9 (13.2)	4 (6.0)
Asian	3 (2.2)	1 (1.5)	2 (3.0)
Hispanic or Latino	74 (54.8)	34 (50.0)	40 (59.7)
White	34 (25.2)	18 (26.5)	16 (23.9)
Multiracial or other^b^	11 (8.1)	6 (8.8)	6 (9.0)
Unknown	5	2	3
BMI *z* score, mean (SD)	2.28 (0.80)	2.25 (0.77)	2.31 (0.85)
BMI percentile, mean (SD)	97.32 (3.05)	97.41 (2.33)	97.22 (3.64)
BMIp95, mean (SD)	122.23 (23.43)	121.54 (22.75)	122.92 (24.34)

Abbreviations: BMI, body mass index (calculated as weight in kilograms divided by height in meters squared); BMIp95, BMI as a percentage of the 95th BMI percentile; FBT, family-based behavioral treatment; PT, parenting training.

^a^ Data are presented as number (percentage) of participants unless otherwise indicated.

^b^ “Other” was an option when participants self-selected their race and ethnicity. No further detail was provided.

^c^ Comprehensive General Parenting Questionnaire: mean self-reported score (range of 1-5, with higher scores indicating parents demonstrated a higher level of the parenting behavior) for each domain of general parenting style.

### Changes in Child Weight Status

**Figure 2.**
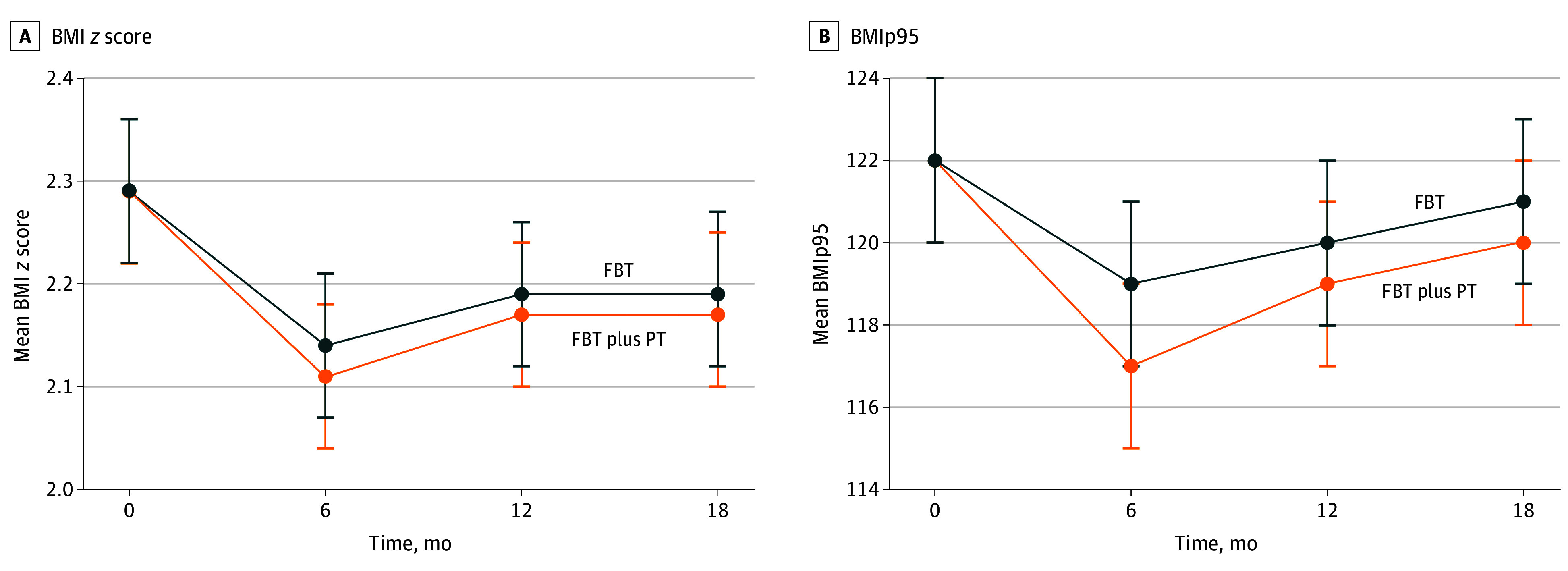
Changes in Body Mass Index (BMI) *z* Score and BMI as a Percentage of the 95th BMI Percentile (BMIp95) After Treatment and at 6-Month and 12-Month Follow-Up Error bars indicate 95% CIs. FBT indicates family-based behavioral treatment; PT, parenting training.

Outcome assessments were completed for 56 FBT participants (80.0%) after treatment, 59 (84.3%) at 6-month follow-up, and 56 (80.0%) at 12-month follow-up and for 59 FBT plus PT participants (84.3%) after treatment, 63 (90.0%) at 6-month follow-up, and 61 (87.1%) at 12-month follow-up. Groups did not differ significantly after treatment or at any follow-up time point (*F*_4, 552_ = 0.12; *P* = .97) (Figure 2, Table 2, and eTable 3 in Supplement 2). When examining combined treatment effects, there was a significant decrease in weight status after treatment in both groups (BMI *z* score: β, −0.14 [95% CI, −0.21 to −0.07]; *P* < .001; BMIp95: β, −3.46 [95% CI, −5.41 to −1.51]; *P* < .001). While both groups had an increase in weight status in the follow-up period, BMI *z* score was still lower than baseline at 12-month follow-up (BMI *z* score: β, −0.08 [95% CI, −0.15 to −0.01]; *P* = .02; BMIp95: β, −1.24 [95% CI, −3.19 to 0.70]; *P* = .20).

**Table 2.** Repeated-Measures Model Parameter Estimates for Child Weight Status Over Time

Measure	Intercept (SE)	β (95% CI)^a^		
		Group	Time	Group × time
BMI *z* score				
6 mo	0.09 (0.06)	0.00 (−0.09 to 0.09)	−0.14 (−0.21 to −0.07)^b^	−0.03 (−0.13 to 0.07)
12 mo			−0.09 (−0.16 to −0.02)^c^	−0.02 (−0.12 to 0.08)
18 mo			−0.08 (−0.15 to −0.01)^d^	−0.02 (−0.12 to 0.08)
BMIp95				
6 mo	2.70 (2.95)	0.03 (−2.7 to 2.8)	−3.46 (−5.41 to −1.51)^b^	−1.72 (−4.48 to 1.03)
12 mo			−1.81 (−3.76 to 0.13)	−1.39 (−4.14 to 1.36)
18 mo			−1.24 (−3.19 to 0.70)	−1.36 (−4.12 to 1.39)

Abbreviations: BMI, body mass index; BMIp95, BMI as a percentage of the 95th BMI percentile.

^a^ Adjusted parameter estimates from mixed-effects regression models with multiple-level multiple imputation reflecting differences between groups (family-based behavioral treatment plus parenting training vs family-based behavioral treatment only) after treatment (month 6), at 6-month follow-up (month 12), and at 12-month follow-up (month 18). All models included planned covariates of race and ethnicity and baseline weight status for child and parent.

^b^ *P* < .001.

^c^ *P* < .01.

^d^ *P* < .05.

### Factors Associated With Clinically Meaningful Weight Loss

Significantly more children receiving FBT plus PT compared with FBT attained clinically meaningful weight loss (reduction of ≥0.20 BMI *z* score units) after treatment (34 [48.6%] vs 22 [31.4%]; *P* = .01); the adjusted odds ratio (AOR) of attaining clinically meaningful weight loss after treatment was 2.10 (95% CI, 1.01-4.47; *P* = .049) (Table 3). In the multivariable model, African American or Black, Asian, and multiracial children had decreased odds of attaining clinically meaningful weight loss compared with White children (AOR, 0.24 [95% CI, 0.08-0.70]; *P* = .01), but there was no difference for males compared with females (AOR, 0.55 [95% CI, 0.25-1.20]; *P* = .14). There were no differences between groups in maintaining clinically meaningful weight loss over time.

**Table 3.** Factors Associated With Attaining Clinically Meaningful Weight Loss After Treatment^a^

Factor	AOR (95% CI)^b^	*P* value
**Parent**		
Age	1.00 (0.94-1.07)	.93
Sex		
Female	0.55 (0.11-2.74)	.46
Male	1 [Reference]	NA
Educational level		
Some college or more	1.47 (0.42-5.58)	.55
≤High school diploma	1 [Reference]	NA
Ethnicity		
Hispanic	1.28 (0.46-3.53)	.63
Non-Hispanic	1 [Reference]	NA
BMI, per unit increase	1.09 (1.01-1.18)	.02
**Child**		
Age	0.79 (0.60-1.04)	.10
Sex		
Female	0.55 (0.25-1.20)	.14
Male	1 [Reference]	NA
Race and ethnicity		
African American or Black, Asian, or multiracial	0.24 (0.08-0.70)	.01
Hispanic	0.39 (0.10-1.45)	.16
Non-Hispanic White	1 [Reference]	NA
Treatment		
FBT	1 [Reference]	NA
FBT plus PT	2.10 (1.01-4.47)	.049

Abbreviations: AOR, adjusted odds ratio; BMI, body mass index; FBT, family-based behavioral treatment; PT, parenting training.

^a^ Clinically meaningful weight loss was defined as a decrease of at least 0.20 BMI *z* score units during the 6-month intervention period.

^b^ Adjusted for age, sex, educational level, race and ethnicity, and parent’s baseline BMI.

### Attendance, Satisfaction, and Adverse Events

Over half of the parent-child dyads (38 [54.3%] in FBT and 37 [52.9%] in FBT plus PT) attended at least 14 sessions. There was no difference in the risk of dropout between the intervention and control groups (hazard ratio, 1.01 [95% CI, 0.72-1.43]; *P* = .95) (eFigure in Supplement 2). In addition, there were no associations between demographic variables and risk of dropout. Treatment satisfaction and acceptability were also similar between groups. Mean (SD) satisfaction scores indicated that parents from both groups thought their program helped their child change behaviors (3.80 [0.88]) and provided useful information (4.27 [0.69]). Parents particularly found the feedback from their behavior coach to be helpful (4.26 [0.84]). Overall, parents were satisfied with the program (4.18 [0.75]) and would recommend it to another family (4.36 [0.87]). There were no serious adverse events in either treatment arm.

## Discussion

To date, few studies have evaluated the effect of intensive parenting skills training on weight loss in children^48^ or have adapted parenting programs to include behavioral lifestyle treatment. While FBT is an effective IHBLT program for childhood obesity,^49^ not all families respond to this treatment; complementary or supplemental treatment modalities may be needed to enhance outcomes. The goal of this trial was to examine the effect of adding a more intensive parenting skills training program for pediatric weight management to traditional FBT and compare it with FBT alone. Parenting training was added to FBT because it has been shown to have an effect on children’s weight even when it is not combined with lifestyle interventions.^50^ While there were no significant changes in weight status between groups, there were significant decreases in child BMI *z* scores and BMIp95 in both groups. Additionally, there was some persistence of effect, which may be due to parent adoption of new lifestyle behaviors. Furthermore, more children in the FBT plus PT group attained clinically meaningful weight loss^24^ after treatment. The addition of more intensive parenting skills training did not affect attendance or satisfaction. Overall, it appears that enhancing parenting skills training may be useful for some families.

While children in the FBT plus PT group were more likely to attain clinically meaningful weight loss, it is still unclear which components of the parenting program were critical to this success. Furthermore, African American or Black, Asian, and multiracial children were less likely to achieve this goal compared with White children, but there were no differences by sex. A recent metanalysis examining sex differences among children enrolled in a behavioral lifestyle intervention also did not find a statistically significant difference in treatment response by sex,^51^ although adolescent boys may respond better to some treatments.^52,53^ Similarly, other studies have demonstrated that weight loss does not vary by race or ethnicity.^54^ Because of the wide variance in weight loss outcomes, it may be statistically difficult to detect differences between sexes and racial and ethnic groups. While we were able to detect subtle differences in treatment response, studies are often not able to capture the phenotypic variation between children and families in a way that allows researchers to understand who responds better to certain treatment types and what might be causing this variability. Further work should be done to examine individual responses to parenting interventions and whether changes in parenting skills or other behavioral factors may mediate changes in weight status.

### Strengths and Limitations

Strengths of this study include the randomized clinical trial design, the diversity of participating families, and the long follow-up period. Limitations include that this trial was impacted by the pandemic, thus reducing the sample size and altering the delivery format. Height and weight measurements were also conducted remotely during the pandemic, and the impact of the pandemic on the study outcomes is unknown. Furthermore, study participants were treatment seeking with a BMI less than the 99.9th percentile; results may not be generalizable to non–treatment-seeking families with higher weight status.

## Conclusions

In this randomized clinical trial, we found that both FBT and FBT plus PT effectively decreased child weight status with no difference in weight status change between groups. More families receiving the enhanced treatment were able to reach clinically meaningful weight loss after treatment compared with those receiving FBT. Identifying which families would benefit from additional PT may allow for an increase in the number of families who are effectively treated. This information may add to the ability to test different combinations of treatment types in a multiphase optimization strategy or sequential multiple assignment randomized trial design^55^ and help create more adaptive and potentially effective interventions that are tailored to specific parent and child needs.

## Notes

Supplement 1.
Trial Protocol

Supplement 2. **eTable 1.** Timeline of Treatment Cohorts **eTable 2.** Content of FBT and FBT Plus PT Treatment Sessions **eTable 3.** Child Weight Status by Group at Baseline, Posttreatment, and 6- and 12-Month Follow-up **eFigure.** Probability of Attrition in FBT vs FBT Plus PT During the 6-Month Intervention

Supplement 3.
Data Sharing Statement
