# Error in Byline

**DOI:** 10.1001/jamanetworkopen.2025.20712

**Published:** 2025-06-11

In the Original Investigation titled “Parenting Training Plus Behavioral Treatment for Children With Obesity: A Randomized Clinical Trial,”^1^ published May 5, 2025, Dr Patel’s academic degree in the byline was changed from PhD to PsyD. This article has been corrected.^1^
