# Supplementary material for: Parenting Training Plus Behavioral Treatment for Children With Obesity: A Randomized Clinical Trial
Source: JAMA Netw Open. 2025 May 5;8(5):e258398. doi: 10.1001/jamanetworkopen.2025.8398 (PMC12053569; doi:10.1001/jamanetworkopen.2025.8398)
Supplement: Supplement 1. — Trial Protocol [file jamanetwopen-e258398-s001.pdf]

UCSD Human Research Protections Program
New Biomedical Application
RESEARCH PLAN

Instructions for completing the Research Plan are available on the HRPP website.
The headings on this set of instructions correspond to the headings of the Research Plan.
General Instructions: Enter a response for all topic headings.
Enter "Not Applicable" rather than leaving an item blank if the item does not apply to this project.

Version date: 05/11/2011

1. PROJECT TITLE

Parent training program to improve outcomes in childhood obesity treatment

2. PRINCIPAL INVESTIGATOR

Kyung Rhee, MD, MSc, MA

3. FACILITIES

1. UCSD, La Jolla Professional Building, 8950 Villa La Jolla Dr., San Diego, CA 92037
2. Center for Community Health, 4305 University Avenue, San Diego, CA 92105
3. San Marcos Civic Center
   1 Civic Center Drive
   San Marcos, CA 92069

4. ESTIMATED DURATION OF THE STUDY

5 years

5. LAY LANGUAGE SUMMARY OR SYNOPSIS (no more than one paragraph)

Authoritative parenting style has been associated with decreased obesity risk in children and can modify the delivery and impact of weight control strategies, making them more effective. At this time, gold-standard treatment for childhood obesity is family-based behavioral therapy (FBT). This treatment relies on behavioral strategies and the use of praise and a positive reinforcement system to change eating and activity behaviors, but does not target other aspects of parenting. Broadening parenting instruction to include effective limit-setting behaviors, parent-child communication, and authoritative parenting may increase parents' confidence and ability to successfully make behavior changes and modify the impact of the behavioral strategies being used. In this application, we propose to test the efficacy of a 6 month, 20-sessions, weight control program that combines traditional family-based behavioral therapy with comprehensive parenting training (FBT+PT) and compare it to traditional family-based behavioral therapy (FBT). We believe that there will be an additive effect of parenting training such that FBT+PT will have a greater effect on child weight loss (measured by BMI z-score) than traditional FBT. In addition we will measure parenting style, parenting strategies, behavioral strategies, and child factors (like impulsive behavior and temperament) to better understand the mediators and moderators of weight loss. These measures will be obtained by standard self-report measures and videotape encounters, allowing us to more objectively measure parenting dimensions. Clinical outcomes, like drop-out and acceptability, will also be assessed from the two treatment arms. The purpose of this study is to evaluate the effect of adding more comprehensive parenting training to traditional FBT. If successful, this program will be able to increase our ability to help children successfully lose weight.

6. SPECIFIC AIMS

Authoritative parenting style has been associated with decreased obesity risk in children and can modify the delivery and impact of weight control strategies, making them more effective. At this time, gold-standard treatment for childhood obesity is family-based behavioral therapy (FBT). This treatment relies on behavioral strategies and the use of praise and a positive reinforcement system to change eating and activity behaviors, but does not target other aspects of parenting. Broadening parenting instruction to include effective limit-setting behaviors, parent-child communication, and authoritative parenting may increase parents' confidence and ability to successfully make behavior changes and modify the impact of the behavioral strategies being used.

In this application, we propose to test the efficacy of a 6 month, 20-sessions, weight control program that combines traditional family-based behavioral therapy with comprehensive parenting training (FBT+PT) and compare it to traditional family-based behavioral therapy (FBT). The FBT+PT arm will incorporate more active

skills learning to increase adoption of authoritative parenting behaviors. We will recruit and randomize 160 overweight/obese children (BMI>85th percentile and <99.9th percentile) between 7 and 12 years old and their parent into a 6 month, 20-sessions FBT+PT or FBT program and follow them for 1 year post-treatment. The primary outcome is change in child body weight (BMI z-score). Our secondary aim will be to evaluate the effect of both treatments on drop-out rates, adherence, and acceptability. As an exploratory aim, we will evaluate parent and child level mediators and moderators of weight loss in each of the treatment arms in order to inform future treatment development. We will achieve the goals of this application by pursuing the following primary and secondary aims:

- **Primary aim 1**: Examine the efficacy of FBT+PT on child body weight compared to gold standard FBT. *We hypothesize that FBT+PT will show greater decreases in child body weight (BMI z-score) compared to FBT at post-treatment and 6- and 12-months post-treatment.*
- **Secondary aim 1**: Examine drop-out, treatment adherence, and acceptability in FBT+PT and FBT. We hypothesize that families randomized to FBT+PT will have lower drop-out rates, increased program adherence (including attendance, self-monitoring, use of praise), and higher ratings of acceptability.
- **Exploratory aim:** Examine mediators and moderators of weight loss in FBT+PT and FBT. We hypothesize that success will be *mediated* by actual change in parenting and behavioral skills, as well as self-efficacy to engage in behavior change. We propose that a) increased parent use of praise, b) effective limit-setting, c) decreased use of harsh parenting techniques, and d) increased behavior change self-efficacy will *mediate* the impact of the intervention on child weight status. We also hypothesize that a) sociodemographic factors (race/ethnicity, income, and education), b) baseline general parenting style, c) parent mental health status, and d) child responsivity to food cues will *moderate* treatment effects. Several factors (e.g., family functioning, chaos level, and child internalizing/externalizing behaviors) may act as both mediators and moderators of treatment effect and will be tested in both capacities.

In September 2017, we received an ancillary grant (R01DK114794), to examine reward and inhibition aspects of cognition, in both parents and children enrolled in this study (R01DK108686), in order to understand the factors that contribute to lack of response in family-based behavioral weight loss. The primary aims of the ancillary study are to determine behavioral and psychological phenotypes of reward and inhibition at baseline in parents and children and to evaluate whether the behavioral and psychological phenotypes at baseline predict weight loss in both parents and children at the 6-, 12- and 18-month timepoints. An exploratory aim is to evaluate whether the behavioral and psychological phenotypes identified at baseline in parents and children change over the measurement period and whether these changes are associated with weight loss.

7. BACKGROUND AND SIGNIFICANCE

The current gold standard treatment for childhood obesity is family-based behavioral therapy (FBT).[1] This program provides the basic building blocks for weight management, including behavior therapy skills and nutrition and physical activity (PA) education. Children in these programs have shown a mean BMI z-score change of -10.3% (S.D. 9.2).[2] However, not all children respond similarly to FBT, with only about a third of children able to maintain their weight loss over a ten year period.[3,4] In addition, programs are often plagued by high drop-out rates, from 20% in academic research settings to 70% in clinical settings,[5] impacting their efficacy. Therefore, studies are needed to determine how to optimize FBT for a greater proportion of families.

Cohort studies suggest that authoritative parenting, characterized by greater warmth/attachment and firm limit-setting, is associated with better weight outcomes.[6,7] This authoritative parenting style may be important because it can alter the context in which behavioral strategies are implemented, thereby making them more effective.[8,9] Furthermore, the use of praise has been associated with greater weight loss in the context of FBT,[10] while permissive parenting is associated with poorer weight loss outcomes.[11] Current FBT relies on behavioral strategies like self-monitoring, stimulus control, and problem-solving, but offers limited instruction on parenting, focusing only on the use of praise and positive reinforcement. Adding more comprehensive parenting training (that includes limit setting strategies and parent child communication skills) to FBT may increase the effectiveness of these programs. Ultimately, parents may become more confident to deliver weight control strategies and be able to do so in a more effective manner.

Parent training programs that focus on improving parent management techniques and parenting style have been successful at increasing parent use of supportive parenting and positive reinforcement techniques, decreasing harsh or permissive parenting, and increasing effective use of limit setting and other discipline strategies.[12-15] Recent data suggest that a parenting program focused on weight loss can decrease child BMI z-score by 0.11 units in an Australian sample.[16] However, this program offered little behavioral therapy and has never been compared to traditional FBT. At this time, it is unclear whether adding more comprehensive parenting training to traditional FBT can increase changes in child body weight compared to traditional FBT.

8. PROGRESS REPORT

N/A

9. RESEARCH DESIGN AND METHODS

Research design: We will enroll an estimated 350 parent-child dyads to treat at least 160 parent-child dyads and randomize them into a 6 month, 20 session traditional FBT or FBT+PT. Families will be followed for 12 months post-treatment, making the total length of participation 18 months. Randomization will be conducted with a random number generator independent of the investigators. All children will be randomized by gender and randomization will be monitored throughout the proposal to determine whether any systematic differences in demography (age, race/ethnicity) or BMI% become evident. Treatment will take place at CHEAR in La Jolla or San Marcos, or remotely via password protected ZOOM meetings. Families randomized to FBT will take part in traditional 20-session group therapy for children and parents that includes nutrition/PA education as well as behavioral therapy components. Groups will include didactic sessions and problem solving. Families who are randomized to FBT+PT will participate in a 20 session program that focuses on teaching positive discipline strategies and effective parenting skills in addition to the components covered in traditional FBT. The process in these groups will include videotape modeling, role plays, and rehearsal (active skills training). Child groups will cover nutrition/PA information and basic behavioral strategies. Since the main difference between FBT+PT and FBT is the parenting training component, and it is not appropriate to introduce parenting issues to the children, the content of child groups will not differ between treatment arms. Once treatment is complete, families will be followed for one year post-randomization. Assessments will occur at baseline, post-treatment (6 months), and 6- and 12-months post-treatment/follow-up. Assessments will include height and weight data obtained during the study period, self-report measures, and videotaped family meals. All assessments that need to be conducted via virtual meetings will be conducted using password protected ZOOM meetings.

Treatment elements: Both treatment arms will consist of 20 weekly group meetings for parents and children. Parent group meetings will last 90 minutes each week and discuss principles of behavior change, nutrition and physical activity. Children will also meet in a separate group at the same time and discuss nutrition and physical activity topics that correspond to the parent meetings. Both parents and children will receive weekly manuals and activities to do at home. The purpose of the manual is to provide key components of family-based behavioral treatment to the families and reinforce what is being taught in groups. Child manuals will provide similar content as the parent manual but be developmentally appropriate for children age 7-12 years old. Parents will also be asked to meet daily with their child to discuss/monitor eating and activity behaviors and other aspects of the program. Parents will be expected to report back on their experiences the following week.

Additional parenting strategies will be addressed in FBT+PT and active skills training used to reinforce these behaviors and parenting style. The parenting training component will be adapted from The Incredible Years Program (IYP).[17] This program is based on social learning models[18] of parent-child interaction and encourage parents to use emotionally supportive behavior and assertive discipline techniques (rather than coercive or negative reinforcement techniques) when interacting with and educating their children. Parents learn how to *use positive reinforcement strategies, use more effective discipline strategies, improve communication techniques, learn how to monitor child behaviors, regulate their own emotions, and problem solve*. This program uses active skills training (*e.g. video modeling, role play and behavioral practice in class, group discussions and problem-solving, and emotion regulation training*) to increase skills adoption.[19,20] Using these techniques, parents can observe others interacting with children in ways that promote positive behaviors

and can practice "doing it right". This method allows parents to gain greater insight into the impact of their behaviors, receive immediate feedback, has been shown to be effective at producing behavioral changes,[21,22] and is not currently used in traditional FBT.

During the first half of each session in FBT+PT, group leaders will address basic behavioral, nutrition, and PA information similar to what is addressed in FBT. Parenting strategies will be introduced and practiced during the second half of the session. Two-minute parenting skills vignettes that demonstrate positive and negative parent-child interactions will be shown by group leaders and discussed with parents. These vignettes have been developed by IYP and are available for purchase. Vignettes that model parents interacting with their child in food and PA situations will be emphasized. After viewing each vignette, parents will discuss what they have seen and practice the targeted skills, receiving feedback from group leaders and other parents. All parents will be expected to role-play during group to help increase mastery of the parenting skills. Parents will problem solve with the group while also learning how to self-manage their thoughts, emotions, and behaviors. Group leaders will reiterate the principle parenting technique that is being addressed and discuss how to use these techniques in different family situations to help increase generalization of the behavior. After each session, parents will also be given assignments to practice these skills at home. At the end of these types of programs, parents often feel more competent to engage in these newly practiced parenting behaviors and learn how to self-monitor their emotions and behaviors, cope with challenges, and seek appropriate support when needed. This more interactive format has been successful at decreasing the use of harsh discipline and critical statements and increasing supportive parenting, displays of affection, and praise.[12,14,15]

Quality Control for Treatment: Close monitoring through multiple methods will be used to ensure treatment integrity. Interventionists (FBT+PT, FBT) will attend full day trainings with Drs. Rhee, Boutelle and Bernard. All interventionists will meet weekly with a licensed clinical psychologist in separate supervisions. Intervention sessions will be audiotaped for ongoing performance monitoring. Random samples (e.g., 20% or more) of all intervention sessions will be rated for fidelity by an independent rater using a measure created for this study. Tapes will be destroyed within three years of completion of the study.

Measures:

Data from the child and the parent will be obtained specifically for research purposes. Data will be collected at 4 time points (baseline, post-treatment (6 months), and 6- and 12-months post-treatment). Assessments will be completed either in-person at the CHEAR office or in a password protected ZOOM meeting. The baseline assessment will include 3 approximately three-hour visits for the child and parent. At the baseline assessment, parents and children will complete surveys and be weighed and measured. Children will also wear an accelerometer for 7 days. Parents and children will be scheduled to complete a videotape family meal at their home. Following the first assessment visit, parents will be given the option of completing some surveys via the secure-web-based survey format prior to attending the in-person visit to reduce the amount of time they need to be present at CHEAR. If parents do not complete the surveys in advance, they will complete them as part of the visit. All surveys provided to be completed at home will include information that does not warrant immediate follow-up (i.e., no questions about suicidal ideation or self-harm). All surveys will be identified with the unique study ID rather than any identifying information. The assessments at follow-up time points (post-treatment (6 months), and 6- and 12-months post-treatment) will be similar to the baseline assessments and will consist of 2 approximately three-hour visits for the child and parent. In addition, families be offered the option of one additional assessment visit (similar to baseline) at post-treatment (6 months), and 6- and 12-months post-treatment, in order to evaluate the aims of the ancillary study. This visit will involve an additional consent form and additional compensation.

Weights will be obtained throughout the treatment program and heights will be obtained at the mid-treatment, post-treatment, and 6- and 12-months post-treatment assessment. The full battery of surveys, accelerometer data, dietary questions, and videotape assessment will occur again at post-treatment and 6- and 12-months post-treatment. A saliva sample for genetic material will only be obtained at baseline. A stool sample for microbiome analysis will be obtained at all time points. A full list of measures and assessments are listed in table 1.

a) *Anthropometry*. Both child and parent will have their body size measured. Height will be measured using a portable Schorr height board in duplicate; body weight will be measured on a Tanita Digital

Scale (model WB-110A) in duplicate. The average of the two values will be used for analysis and will be translated for children to BMI for age percentile score using the CDC growth charts and to Body Mass Index(BMI) in parents (BMI=[kg/m2]). Children with a BMI ≥ 85th percentile and parents with a BMI ≥ 25 will be considered overweight.

b) *Dietary intake*. Dietary intake of the child will be assessed with dietary knowledge questionnaire, which will assess eating habits of the children and the parents.

c) *Physical activity.* Physical activity of the child and parent will be assessed using Actigraph accelerometers. Actigraph accelerometer (model GT1M, www.theactigraph.com) is a small (3.8 x 3.7 x 1.8 cm), lightweight (27 g), uni-axial accelerometer that is worn on a belt snugly around the waist. The GT1M records time varying accelerations ranging in magnitude from approximately .05 to 2 G's. We plan to ask participating children to wear the Actigraph for 7 consecutive days so that we may be able to assess within-person variation more precisely than if we collected data for only 4 days. Trained staff will instruct participating children and their parents on the proper procedure for attaching the Actigraph to their waistband and completing a log to record the times they did not wear the Actigraph (they'll be asked to remove the actigraph during sleeping time).

d) *Sedentary behaviors & recreational media use.* Sedentary behaviors and media use will be assessed using an instrument developed in the PACE+ Adolescent trial. Both child and parent will complete this measure. This 8-item survey was based on a survey developed by Robinson and measures hours spent doing various sedentary behaviors during a typical school day and non school day. Analysis of the survey indicated that 3 factors account for 58% of the variance. The three factors were "Idle" (TV, computer/video games, listening to music, talking on the phone), "Intellectual" (homework, reading), and "Creative" (playing musical instrument, arts and crafts).

e) *Activity logs.* In addition to wearing the actigraph for 7 consecutive days, children and parents will complete activity logs that include physical activity and sedentary activity. Children will record their activity, television usage and computer usage on specific activity logs for the assessment. When children and parents receive instructions on using the actigraph, they will also receive instructions on completing activity logs. Actigraphs's and activity logs will be returned to project staff at the first treatment session.

f) *Demographics characteristics and Weight History (Baseline only).* Parent and child will complete separate demographic questionnaires that includes items on personal weight history, types of weight management strategies, dieting, weight fluctuation, health history, and parents will complete a locator information for assistance in tracking individuals.

g) Videotape measures will be used to obtain objective measures of parenting. Families will be videotaped eating a meal together, in their home, at baseline, post-treatment, and follow-up. Only immediate family members living within the household will be invited to participate in this recording, and no additional guests will be allowed. The research team has experience collecting data in this method and has a standardized protocol for the taping and coding of **mealtime feeding behaviors** (see Appendix). This schema captures behavioral therapy strategies such as portion control, stimulus control, modeling, and monitoring used in a feeding situation. Additional coding schemas to be use are the ***Coder Impression Inventory (CII)***[14,23] and the ***Mealtime Family Interaction Coding System (MICS).***[24] The data from these tapes will be used for research purposes only. Only Dr. Rhee, her trained research staff and collaborators will have access to these de-identified tapes.

h) *Executive functioning (EF: child and parent).* *Attentional control, cognitive flexibility, working memory, and general cognitive abilities* will be measured by the Flanker Inhibitory Control and Attention Test, Dimensional Change Card Sort Test (DCCS), Picture Vocabulary Test, Picture Sequence Memory Test, Verbal List Learning Task with Food, Digit Span, Attention Bias Task, Relative Reinforcing Value of Food, and Oral Reading Recognition Test. *Inhibition* to food and non-food cues will be measured by the *stop signal task-food version*. On each trial, participants are asked to discriminate between a picture of calorically-dense food or neutral object (e.g, chair). The two primary outcomes, stop signal reaction time for food pictures (SSRT-food) and neutral pictures (SSRT-neutral) will be compared to assess for food-specific impulsivity versus general impulsivity. *Overall child EF* will be measured by the Behavior Rating Inventory of Executive Function – Parent Report (BRIEF-2), which is completed by the parent. This 63-item form contains three indices, nine clinical scales, and three validity scales. *Overall parent EF will be measured by the* Behavior Rating Inventory of Executive Function—Adult (BRIEF-A), to be completed by the parent. This 75-item form contains nine clinical scales and three validity scales.

i) *Psychophysiological Measurements.* All of the psychophysiological measures were chosen as measures

of Cephalic Phase Responses (biological preparatory responses to food) and have shown sensitivity to conditioning paradigms with food. Cephalic Phase Responses (CPRs) have been associated with responses to food and perceived craving in humans1 and differentiate overweight and normal weight adults.4 Electrophysiological recordings will all be sampled at 250 Hz. Participants will be exposed to highly palatable food for 6 minutes and a neutral object for 2 minutes.

j) *Parenting (child and parent).* A component of the study assessment procedures will include a battery of reliable and valid measures of parenting, including The *Child Report of Parent Behavior Inventory,*[25] the *Comprehensive Feeding Practices Questionnaire,*[26] and *The Consideration of Future Consequences Scale,*[28] *Comprehensive General Parenting Questionnaire, Birch Child Feeding Questionnaire, Parent Importance Confidence and Readiness Questionnaire, Parent Motivation Inventory, Family Adaptability and Cohesion Scale IV*.

k) *Treatment adherence (child and parent).* Adherence to recommendations (e.g. goal setting, stimulus control, contracting) and self-monitoring (% days monitored) for child and parent will be collected using standard study assessment tools to assess treatment adherence. All self-monitoring will be scored for number of days that participant monitored any meals or snacks and for number of days that participants monitored all meals or snacks (as defined by three, time distinct entries for meals and at least one snack). Parents and children will also complete treatment adherence questionnaires that query how many days they self-monitored immediately after meals, how many days they self-monitored at the end of the day, and how accurate they perceive their own self-monitoring to reflect actual intake.

l) *Treatment acceptability (child and parent).* Questionnaires will assess parent and child liking and any concerns or barriers to implementing the gshFBT or the FBT treatment.

m) *Gut microbiome*: Participants (parent and child) will be sent a stool collection kit and asked to provide a stool sample within 24 hours of their pre- and post-treatment assessment visits. The BBL CultureSwab will be used to collect samples from subjects (Becton-Dickinson catalog #BD281130) from tissue paper. These swabs have been used successfully by the Knight lab, who has shown that microbiome analysis using these swabs was comparable to that using more traditional stool collection methods. De-Identified stool samples will be stored at -20°C. Microbiome analysis will be conducted to examine the relationship between changes in weight and gut microbiome.

n) *Genetics.* Parent and child will each give a saliva sample which will be collected using the Oragene•DNA saliva collection system. Samples will be collected before the first treatment session. Saliva will be obtained, stored at room temperature using the deidentification number assigned to each patient, and banked for later analysis. Genetic data will be used to examine the relationship between target SNPs and eating behaviors. Disease specific susceptibility genes will not be examined. Data is for research purposes only and subjects will not be informed of the results of their genetic testing.

Analysis:

Analyses will be based on linear mixed models implemented in R statistical software and Mplus v 6.12. Primary outcomes evaluation first will follow an Intention to Treat principle with all missing values presumed returned to baseline level. Planned covariates for primary outcome, change in BMI z-score, will include gender, ethnicity, and parent marital status. For models evaluating the Primary Aim, fixed effects will include coefficients for time, treatment, and time by treatment interactions. For each outcome, selection of a covariance structure for the residuals will be based on fitting different structures (e.g., compound symmetry, unstructured) and selecting the one with the lowest sample size adjusted Akaike's Information Criterion.[25] Significance tests will focus on the treatment by time interactions.

We will conduct analyses examining patient and therapist ratings of treatment acceptability and whether participants believe that the FBT+PT or FBT components address issues that are particularly relevant to them given the level of child behavioral problems. These analyses for the Secondary Aim will be conducted using regressions comparing FBT+PT to FBT. Survival analysis will assess the effect of FBT+PT on decreased risk for treatment drop-out (defined as subject refusal to continue treatment or failing to return to treatment without notification). Planned covariates in survival models will mirror primary analyses. Exploratory analyses will include use of generalized linear mixed effects models for repeated assessments of limited categorical and binary outcomes reflecting energy balance, video tape data, parenting, feeding/lifestyle behaviors, and psychosocial factors.

A multiple-mediator model[19] will provide a test of whether FBT+PT leads to greater changes than FBT on proposed mediators ('a' paths) and whether changes in mediators (e.g., use of praise, limit-setting, problem-

solving, self-monitoring) at the end of treatment are related to greater decreases in BMIz over time ('b' paths) (Exploratory Aim). The product of these sets of coefficients[22] and associated standard errors[19] will provide effects used to test statistical significance. Mplus 6.12 includes both normal theory and bootstrap methods for estimating both total indirect (all mediators) and specific indirect effects for each proposed mediator. For the moderator hypotheses tested as part of the Exploratory Aim, the models used for the primary aim will be augmented by including a coefficient for the main effect of the moderator (e.g., parent mental health, baseline general parenting style, child food-cue responsiveness) and interactions with treatment and time.

For the Ancillary study:

Primary aim 1: To determine behavioral and psychological phenotypes of reward and inhibition at baseline in parents and children: Latent Profile Analysis[26] (LPA) will be used to derive empirically defined groupings of participants based on common patterns of responses to hypothesized inhibitory control (behavioral motor, emotional regulation, and memory for food), reward function (normed physiologic reactivity, self-reported reward responsiveness), and cognitive bias (attentional bias, Stroop bias) indices of risk via maximum likelihood estimation. Models will be estimated with numbers of classes determined iteratively using the Lo-Mendell-Rubin Adjusted Likelihood Ratio Test[27] (LMRT), the Bootstrapped Likelihood Ratio Test[28] (BLRT), Akaike information criteria[29] (AIC), and sample size-adjusted Bayesian information criteria[30] (BIC). In addition to these indices, each model will be evaluated using interpretability of identified conditional means to protect from spurious classes based upon nonnormal distributions.[31]

Primary Aim 2: To evaluate whether the behavioral and psychological phenotypes at baseline predict weight loss in both parents and children at the 6-, 12- and 24-month timepoints: Generalized Linear Mixed Effects Model (GLMM) regressions to accommodate potentially non-normal error distributions with random intercepts will evaluate 0-, 6-, 12-, and 24-week post-treatment BMI(z) for parents and children. Linear and non-linear effects for changes in BMI(z) will be examined for inclusion using evaluation of fit for competing unconditional models. Planned covariates will be added to include age and gender. Main effect terms for the treatment types (dummy coded with guided self-help FBTas reference) will be estimated and tested as a set. The set of baseline risk profile membership scores from LPA will be examined in a series of generalized LME models to assess independent main effects on changes over time (e.g. slopes) and potential moderating effects of treatment on changes in weight (e.g. three-way interactions). Given a potentially large number of predictors proposed to assess child and parent phenotype interactions

Exploratory aim 1: To evaluate whether the behavioral and psychological phenotypes identified at baseline in parents and children change over the measurement period and whether these changes are associated with weight loss: We will use in GLMM to explore changes in inhibitory control functions, reward function, and cognitive bias indices for children and parents over 6-, 12-, and 24-month assessments. We will evaluate whether the set of baseline phenotype indicators are associated with observed changes in these measures with adjustment for planned covariates and corresponding baseline values separately for children and parents.

Table 1: Measures	Instrument	Child or parent	Base-line	Post-tx (6 month)	6 month Follow-up	12 month Follow-up
**Anthropometry**	Height and weight	C, P	X	X	X	X
**Energy Balance Behaviors**	Dietary Knowledge Questionnaire	C	X	X		X
	DHQ-II	P	X	X		X
	Accelerometers and actigraphy	C	X	X		X
	Global Physical Activity Questionnaire[32]	P	X	X		X
**Demographics**	Race, medical history, weight history	P	X			
	SES-Barratt Simplified Measure of Social Status (Hollingshead modified)[33]	P	X			
	US Household Food security module	P	X	X	X	X
	Exercise Environment Questionnaire[34]	P	X	X	X	X
	Social support[35,36]	C, P	X	X	X	X
	CHAOS in the home[37]	P	X	X	X	X
**Videotape data**	Mealtime feeding behaviors	C, P	X	X	X	X
	Coder Impression Inventory	C, P	X	X	X	X
	Mealtime Family Interaction Coding System[24]	C, P	X	X	X	X
**Parenting**	Child Report of Parent Behavior Inventory[38,39]	C	X	X	X	X
	"Getting Along with My Parent" questionnaire[40]	C	X	X	X	X
	Raising Children Questionnaire.[41]	P	X	X	X	X
	The Parenting Scale[42]	P	X	X	X	X
	Parent Importance, Confidence, & Readiness questionnaire[43]	P	X			
	Parent self-efficacy for health-related behaviors[44]	P	X	X	X	X
	Lifestyle Behavior Checklist (Confidence scale)[45]	P	X	X	X	X
	Parenting Stress Index-Short form[46,47]	P	X	X	X	X
	Family Adaptability and Cohesion Scale IV	P	X	X	X	X
**Feeding and lifestyle behaviors**	Child Feeding Questionnaire[48]	P	X	X	X	X
	Adult/Child Eating Behavior Questionnaire[49],[61]	P, Pc, P	X	X	X	X
	Emotional Eating Scale-Child version[50]	C	X	X	X	X
	Reward-based Eating Drive-13[51]	C,P	X	X	X	X
	Eating Disorder Evaluation (binge eating questions)[52]	C	X	X	X	X
	Lifestyle Behavior Checklist (Problem Scale)[45]	P	X	X	X	X
	Parent Mealtime Action Scale[53]	P	X	X	X	X
	Family Eating and Activity Habits Questionnaire[54]	P	X	X	X	X
**Psychosocial functioning**	PRIME MD Patient Questionnaire[55]	P	X			
	CES-D[56]	P	X			
	Child Behavior Checklist[57]	P	X	X	X	X
	Pediatric Quality of Life Inventory[58,59]	C, P	X	X	X	X
	MINI	C	X			
	State-trait anxiety inventory	C,P	X			
**Executive Functioning and Cognitive Ability**	WRAT-4	C,P	X	X	X	X
	Barratt Impulsivity Scale	C, P	X	X	X	X
	UPPS-P	C,P	X	X	X	X
	NIH Toolbox Battery	C,P	X	X	X	X
	CVLT-Food	C,P	X	X	X	X
	Food-Stroop	C,P	X	X	X	X
	Relative Reinforcement Value of Food	C,P	X	X	X	X
	Stop Signal Task	C,P	X	X	X	X
	Digit Span	C,P	X	X	X	X
	Attention Bias	C,P	X	X	X	X
	Delay Discounting[60]	C,P	X	X	X	X
	Behavior rating Inventory of Executive Functioning	P,Pc	X	X	X	X
**Biologic Specimens**	Saliva Sample – genetics	C, P	X			
	Stool Sample - microbiome	C,P	X	X	X	X
**Treatment Adherence, Drop-out, Acceptability**	Attendance (weekly assessment)	C, P		X		
	Habit books (self-monitoring) (weekly assessment)	C, P		X		
	Behavioral adherence (developed by team)	C, P		X	X	X
	Drop-out rates	C, P		X	X	X
	Satisfaction survey	C, P		X		

10. HUMAN SUBJECTS

Overall we expect to consent 350 child/parent dyads and have at least 160 child/parent dyads get randomized to treatment who meet the following eligibility criteria. Research involving pediatric obesity interventions typically have a large number of female participants (60%) compared to male participants. Based on previous pilot work completed for overweight/obese children age 4-12 years, more than 70% were female. Therefore, we expect that at least 60% of the participants will be female and 40% male. Our goal will also be to obtain a racial/ethnic breakdown of participants that is similar to that of San Diego County. San Diego encompasses a wide range of ethnically diverse individuals, including 52.9% white non-Hispanic, 27.9% Hispanic, 11.1% Asian-American, 4.7% African-American 1.6% American Indian and Alaska Native, 0.5% Native Hawaiian or Pacific Islander. Considering these statistics, we expect that we will obtain an ethnically diverse sample of San Diegan children.

Inclusion criteria are: a) An overweight child age 7-12, whose BMI is ≥ 85th percentile but <99.9^{th} percentile for age and gender; b) The parent willing to participate and can read English at a minimum of a 5th grade level; c) Parent and Child willing to commit to attending all treatment and assessment sessions and be randomized to either treatment arm; d) Child, parent, and 1st degree relatives are free from psychiatric illness than may affect participation; e) Child does not have any medical conditions that impact weight or may affect participation in physical activity or treatment f) Child is not taking medications that may impact their weight (unless medication dosage is stable and not for the purpose of impacting weight and appetite).

Exclusion criteria include: 1) Children with serious medical conditions that affect their weight; 2) Children taking medication that affect appetite or weight; 3) Children with severe developmental delay or disability that would affect participation; 4) Children or parents with psychological illness that would limit treatment participation; 5) Families who will move out of the area within the time frame of the study.

Prior to inclusion, parents and children will be weighed and measured and BMI calculated to determine if they are eligible for participation. Children must have a BMI percentile that is ≥85th percentile, according to the CDC criteria for age and gender. Parents must have a BMI≥25 to qualify.

11. RECRUITMENT

All advertisements for the intervention will target families with at least one child who they think is overweight/obese in the target age range. Advertisements will state that this is a *family* weight loss program intended to make lifestyle changes that will benefit the whole family. Both parent and child must agree to participate in the study. Children who state that they are not interested in participating in the program will not be enrolled, as well as their parents.

Families will be recruited through advertisements placed in local newspapers, radio, family newsletters, TV ads, ResearchMatch, clinicaltrials.gov, and the internet. If needed, information for direct mailing/emailing will be purchased from companies or services such as the U.S. Postal Service that allow you to purchase/ pay to send mail or email to individuals. Families will also be recruited from Rady Children's Hospital potentially through the use of electronic medical records (EPIC system). A request for partial waiver of HIPAA authorization for recruitment purposes has been submitted and additional approval will be obtained from this institution in order to do so. Additional flyers and posters will be placed in pediatricians' offices, community centers, and schools. We will also recruit potential participants through a "snowball recruitment" effort. This effort would involve current participants in our study (either in the treatment or follow-up phase of the study). These current participants will be asked to refer other potential families that they think would be interested in our study. They will be given previously approved recruitment flyers to give to these new potential families. They will not be compensated for these recruitment efforts or given preferential treatment during the intervention because of these efforts.

For our study, our recruitment staff will use our IRB approved ads and text, landing pages, and online screens to advertise. Our recruitment staff may advertise on Facebook, Google, Instagram, medical informational sites, and other relevant app networks. The ads will target adult parents over the age of 18 with the advertisements in the San Diego area. Once someone responds to an ad, they will be directed to our landing page with information about the study. The language on this landing page is the exact language on our current IRB approved landing page. Interested parents can complete a prescreener that is embedded directly on the landing page with their name, email, phone number, a best time to call, and information that will help us

determine the child's age, home zip code, and BMI percentile to assess basic eligibility for this study.This will register their interest in this study. The information will be sent from a secured webpage hosted by a secure server, through a secured email to a UCSD email address that is only accessed by approved study personnel through a secure UCSD login. Only approved study personnel within the lab will have permission to access provided information so that we may move forward with the screening process.

Participants who respond to all recruitment efforts, including social media advertisements, will be asked to complete an initial online screen to determine initial eligibility. If individuals do not have online access or do not wish to complete the screen online, they can complete an extended screening call that will include these questions as part of the telephone screen. Participants who meet study inclusion criteria will then complete a phone screen to further assess eligibility.

We are requesting a Waiver of Documented Consent for the phone screen because this process meets criteria for minimal risk to the subject. We will ask the participants if they are willing to answer additional screening questions over the phone and obtain verbal consent for this process. This component is minimal risk to the participants because: 1) the waiver will not adversely affect the rights or welfare of the subjects; 2) this component could not be practicably carried out with the waiver; 3) there are no additional risks outside the usual scope of practice in answering these questions; and 4) pertinent information will be provided to the subjects after participation.

If participants meet initial screening criteria, they will be scheduled for an orientation to learn more about the study, review the informed consent and have all questions answered. If they remain interested in participating, they will then sign an informed consent and be scheduled for the first assessment.

12. INFORMED CONSENT

The online screening will assess for basic inclusion and exclusionary criteria. This process presents no more than minimal risk of harm to subjects, and involves no procedures for which written consent is normally required outside of the research context. For these reasons, we request a waiver of documented consent for the online screening. For individuals who do not have online access, the questions asked on the online screen will be administered over the phone in conjunction with the phone screen.

The phone screening will assess for basic inclusion and exclusionary criteria. This process presents no more than minimal risk of harm to participants and involves no procedures for which written consent is normally required outside of the research context. For these reasons, we request a waiver of documented consent for the phone screen.

Those families who appear to be eligible will be invited to the research office or remotely via Zoom for orientation during which time a more complete description regarding the purpose of the research, the timeline and procedures of the study, the risks and benefits, and how confidentiality will be maintained will be provided. Family members will be given an opportunity to ask questions at this time and informed that participation is voluntary and that they can withdraw from the study at any time. The orientation and consent process may take place with a group of potential participants. Participants may ask questions as a group and they will also be informed that they will each have an opportunity to privately ask additional questions. If individuals are unsure as to whether they are willing to participate in the study, they may take the consent form home with them. If they later decide they wish to participate, they may schedule their first assessment and sign the consent form prior to participating in the assessment.

Families who are eligible and agree to participate in the study will then provide parental informed consent and child assent that has been approved by the Institutional Review Board of UCSD after the orientation session. Consent will be obtained by one of the study investigators or the program coordinator. All consenters have or will have completed HIPAA training prior to obtaining consent. At this time, only families comfortable communicating, reading, and writing in English will be enrolled in the study.

For those who are consented in a zoom meeting, we will require a waiver of written consent. During the zoom call, the study staff member responsible for obtaining verbal consent will review the emailed document with the

participant. The study staff member will provide an opportunity for the potential participant to ask questions. When all of the potential participant's questions have been answered, the study staff member will ask the participant if they are ready to provide consent and then verbal consent will be obtained. Should the potential participant desire more time to make a decision to participate, they will be provided information on how to reschedule their zoom verbal consent call. Justification for this oral consent procedure is that the research presents no more than minimal risk of harm to subjects and involves no procedures for which written consent is normally required outside of the research context.

Families who are enrolled in the main study and agree to participate in the optional additional assessments for the ancillary study will complete an additional parental informed consent.

An amendment on May 2, 2019 changed the length of storage for video data. In order to facilitate the re-consenting process for participants who have completed the study prior to the approval of the amendment, the study is requesting a waiver of documented consent for these participants. The study team will make several attempts to contact these study participants, first via phone (x2), then via email (x2), before a letter is sent to the family. These phone calls and emails will ask the family to contact the study team so we can inform them of the changes to the protocol and obtain verbal consent over the phone.

- The changes involve minimal risk to subjects, as they are limited to changes that impact the timeline pertaining to storage of data.
- The changes involve no procedures for which written consent is normally required outside of the research context.
- The verbal consent will be documented by the study team on the originally signed consent form and in a data sheet for tracking purposes. Standard note to file will be added to the subject's chart indicating the date which they agreed to the changes.

If a subject chooses not to agree to the extended storage timelines, the video data will be stored for the length of time to which the subject initially agreed.

In the event that no response is received for the request for consent for these changes in data storage timeline, the study is requesting a waiver of consent to allow for the extended storage of video data for subjects who completed the study prior to approval of the amendment that changed storage timelines.

- The changes to storage timelines represent minimal risk.
- The changes do not adversely impact the rights and welfare of subjects, as they previously agreed to storage of their videos and the amendment simply modifies the time frame. Appropriate confidentiality measures will still be in place to protect the data.
- The research could not practicably be carried out with the waiver because some of the families have completed the study and are no longer in contact with the study team.
- Whenever appropriate, the subjects will be provided with additional pertinent information after participation.

13. ALTERNATIVES TO STUDY PARTICIPATION

Subjects can choose not to participate in the study at any time. There are no alternative therapies provided by the study. If the subject chooses not to participate, he/she can enroll in other clinical weight control programs offered in the area or at Rady Children's Hospital.

14. POTENTIAL RISKS

During the program, participants will be asked to improve overall dietary intake and increase physical activity behaviors. Increasing the consumption of healthy foods while reducing high caloric foods may result in more than expected weight loss. Although increasing physical activity can have great benefits, participants may also experience some general fatigue or sore muscles or joints from being active. It is also possible that participants could fall or be injured in association with being physically active. Most of these injuries are not likely to require medical treatment. Performing moderate intensity physical activity such as walking reduces

the risk of these complications. There is no guarantee, however, that these complications will not happen to the parents and/or children.

Families may also be inconvenienced or frustrated by the assessment process. We will try to minimize any inconvenience for family members by scheduling assessments at their convenience and limiting the amount of time required for these assessments. Videotape family meals will occur in their home at their regularly scheduled mealtime to decrease any inconveniences and increase the validity of these assessments. Children may also become frustrated by or not like the intervention, surveys, or videotaped meals. Children who become frustrated or are experiencing extreme stress during the intervention or assessments will be allowed to stop and withdraw from the study without any consequence to themselves or their parents. Children may also feel uncomfortable being videotaped during assessments. However, most children become accustomed to being recorded after a few minutes.

Should a child or parent display symptoms indicative of physical or mental disorders during treatment, the interventionist will assess the problem, and in consultation with study supervisors, Drs. Rhee, Bernard, and Boutelle, decide whether to withdraw the individual from treatment and/or to refer them for treatment elsewhere.

Safety of human participants is a major concern in a trial involving youth. The initial reporting of adverse events of any kind will take place with the study staff consulting with the PI. The PI will then decide whether the events are of such a severity that requires discontinuation of a particular treatment, and whether the participant should remain in the study or be withdrawn and referred elsewhere. In this study, adverse events will be defined as unanticipated problems involving risks to the study participant. A serious adverse event will be defined as any untoward occurrence that results in death; is life threatening; or creates persistent or significant physical or emotional disability. All such events will be immediately reported to the IRB.

Risk of loss of confidentiality: There is a small risk that confidentially of survey data as well as other biological measurements will be lost or stolen during this study. However, the research team will make every effort to maintain this confidentiality (see details in Section 15). The nature of the genetic and microbiome information to be studied is also of low risk and has minimal chance to impact future employability, insurability, reputation, or other social factors. Disease specific markers will not be examined. The biologic information gathered in this study is for research only and will not be provided to the participant’s physicians, insurers, or employers.

This intervention may also involve risks that are currently unforeseeable to us at this time. There is also a potential risk of loss of confidentiality, which is addressed in the following section.

15. RISK MANAGEMENT PROCEDURES AND ADEQUACY OF RESOURCES

All parents will be advised about healthy eating and activity practices including dietary changes and increasing physical activity/decreasing sedentary behaviors in the group meeting. Parents will be advised to gradually make changes in these behaviors. The child and parent’s weight will be monitored weekly. However, parents may report excessive weight loss in themselves or their children. If this occurs, an increase in caloric intake or encouragement of healthier, alternative practices will be made. Children may be asked to come to the research office so that weight and height can be measured by research staff and discussed with myself, a board certified pediatrician. If unhealthy practices or significant weight loss continues, the child’s pediatrician will be notified and referrals for other treatment made.

Participants will also receive a thorough introduction to the study questionnaires, assessment tasks, and videotaped family meal preparing them for the nature of the questions/activities. Such preparation often decreases the risk of becoming unduly distressed. If a participant does become distressed, study staff will be trained to respond in a supportive, empathic way and to give the subject the opportunity to take a break. The intervention materials will address how to lose weight and develop healthy eating and activity habits, which is generally considered healthful. The consent process will be discussed with the participants, and they will be assured of the confidentiality of their responses.

Confidentiality of participants will be protected in multiple ways. First, all participants will be given a unique identification number that will be used on all documents with no references to individual names, addresses, phone numbers or birthdays. Videotapes will also be given unique identification numbers and coders will be blinded to additional identifiers except for any names mentioned in the videotapes. Subjects may request that the taping be stopped or that part or all of the recording be erased at any time. Subjects may also view the recording prior to submitting it, and be allowed to not submit the data without any consequence to themselves. All data will be stored in locked file cabinets in locked rooms. The de-identified data will be entered into a password-protected computer that only the trained research staff, collaborators, and the PI will be able to access. All procedures will be approved by UCSD's Institutional Review Board to ensure that they meet standards for the protection of human subjects.

There is also the potential risk of loss of confidentiality. However, the research team will make every effort to keep all personal identifying information confidential. A unique identification number will be provided for each participant so that all data can be de-identified. Files linking names with subject ID numbers will be kept in a locked file that only the PI will have access to. All other data will be stored in locked cabinets in locked rooms in UCSD sponsored facilities or on a password protected computer that only the PI and RA will have access to.

Dr. Rhee's lab has well established protocols if a child or parent endorses anything that suggests abuse, neglect or harm to self or others. If any concerning statements are made at any time during the assessment of the family, or during the treatment of the family, Drs. Rhee, Bernard, or Boutelle will be immediately alerted and reviews statements made by the family with the staff member. All child abuse, harm, and neglect cases are reported to the state of California Child Protection Service, as required by the mandated reporting laws. Because of this low risk status, the data and safety monitoring plan (DSMP) for this trial focuses on close monitoring by the principal investigator (PI) in conjunction with a safety officer, along with prompt reporting of excessive adverse events and any serious adverse events to the NIH and to the IRB at the University of California San Diego.

16. PRIVACY AND CONFIDENTIALITY CONSIDERATIONS INCLUDING DATA ACCESS AND MANAGEMENT

Confidentiality of participants will be protected in multiple ways. First, all participants will be given a unique identification number that will be used on all documents with no references to individual names, addresses, phone numbers or birthdays. Files linking names with subject ID numbers will be kept in a locked file that only the PI will have access to. Assessment videotapes will also be given unique identification numbers and coders will be blinded to additional identifiers except for any names mentioned in the videotapes. Subjects may request that the taping be stopped or that part or all of the recording be erased at any time. Subjects may also view the recording prior to submitting it, and be allowed to not submit the data without any consequence to themselves. De-identified video assessments will only be used for research purposes and viewed by trained research staff, and collaborators of Dr. Rhee in future research projects. All data will be stored in locked file cabinets in locked rooms. Data will be entered into a password-protected computer that only the research assistant and PI will be able to access. All procedures will be approved by UCSD's Institutional Review Boards to ensure that they meet standards for the protection of human subjects.

17. POTENTIAL BENEFITS

The potential health benefits associated with decreasing BMI are great. Reductions in BMI, as well as healthy dietary changes and increased physical activity, have the potential to improve long-term cardiovascular, respiratory, endocrine and orthopedic health. Information gained from this study may also help to develop more effective interventions for overweight children in the future.

18. RISK/BENEFIT RATIO

The risks of participating in this program are considered to be minimal. In comparison, the potential health benefits to the subject and the knowledge gained from this study are considered to be significant. Given that

the risks are minimal and the potential for benefit is great, the risk/benefit ratio is considered to be small and reasonable for the conduct of this study.

19. EXPENSE TO PARTICIPANT

There is no cost to the subject for participating in this study. Secondary costs may include travel to and from the research site and childcare for any children not participating in the research study.

20. COMPENSATION FOR PARTICIPATION

All families will receive $25 for each day of the pre-treatment assessments (potentially 3 days equaling $75), $50 for the post-treatment assessment, and $100 each for the 6- and 12-month follow-up assessments as reimbursement for time, travel and effort. An optional additional assessment session will be offered at post-treatment ($50), 6-month follow-up ($100), and 12-month follow-up ($100) in order to collect measures for the ancillary study. All compensation will be in the form of UCSD scrips or gift cards.

21. PRIVILEGES/CERTIFICATIONS/LICENSES AND RESEARCH TEAM RESPONSIBILITIES

Dr. Kyung Rhee is an M.D. with privileges at Rady Children's Hospital. She is trained in the delivery of family-based weight control interventions, parenting programs, and the conduct of clinical trials. She will be conducting the intervention and overseeing the overall project. She has also undergone the mandatory Human Subjects Education Training on 7/29/03, and renewed on 6/30/05, 4/24/07, 9/23/08, 2/18/2010, 7/20/11. She will be responsible for making sure that all other personnel on the team be trained in Human Subjects Research via the CITI Collaborative Institutional Training Initiative.

Dr. Kerri Boutelle is a clinical psychologist with expertise in pediatric weight loss programs and parenting. She will participate in all program activities. She has completed the mandatory Human Subjects Education Training.

Dr. David Strong is a psychologist with expertise in statistical methods. He will participate in study design and data analysis. He has also completed the mandatory Human Subjects Education Training.

Dr. Bess Marcus is the department chair for Family Medicine and Public Health at the University of California, San Diego. She will provide input on the overall direction of the project, specifically in regards to physical activity measurement and intervention.

Dr. Cheryl Anderson is an Associate Professor in the Department of Family Medicine and Public Health at the University of California, San Diego. She is an expert in the field of nutrition, and will contribution her knowledge and training in nutrition, epidemiology, and health behavior and education to this study. She will provide input on the overall direction of the project, specifically in regards to nutrition measurement and intervention.

Dr. Rebecca Bernard is a licensed psychologist at the Center for Healthy Eating and Activity Research that will oversee the fidelity of the intervention and conduct weekly supervision with all interventionists. She may obtain consent.

Dr. Michael Gottschalk is a Professor of Pediatrics at the University of California San Diego and the Division Chair for Pediatric Endocrinology at Rady Children's Hospital. As safety officer for this study, Dr. Gottschalk will review the reports sent by the study coordinator and will determine whether there is any corrective action, trigger of an ad hoc review, or stopping rule violation that should be communicated to the study investigator, the University of California, San Diego IRB, and the funding agency.

Dr. Takisha Corbett has a Ph.D. in clinical psychology and is a postdoctoral fellow. She is the project director. She will assist with project coordination, assessment, and intervention. She will oversee the budgetary and administrative tasks management of the study, including the budget, hiring personnel, personnel

issues, equipment management, NIH compliance and implementation of refinements to the protocols, and may obtain consent.

Dr. Dawn Eichen is a postdoctoral fellow and a licensed clinical psychologist. She will help train interventionists and provide supervision as well as assist with project coordination, assessment and intervention. She will oversee the budgetary and administrative tasks management of the study, including the budget, hiring personnel, personnel issues, equipment management, NIH compliance and implementation of refinements to the protocols, and may obtain consent.

Dr. Eastern King, is a postdoctoral fellow. He will oversee data analyses including evaluating all data entry accuracy, merging of data files, creation of scales, and evaluating initial frequencies and means.

Dr. Saori Obayashi has a Ph.D. in nutrition and a Registered Dietitian. She will assist with project coordination, assessment, and intervention. She will oversee the budgetary and administrative tasks management of the study, including the budget, hiring personnel, personnel issues, equipment management, NIH compliance and implementation of refinements to the protocols, and may obtain consent.

Dr. Shamin Patel is a Staff Research Associate. She will assist with project coordination and intervention and may obtain consent.

Elena Kaltas, MA is an Academic Program Management Officer. She may help with subject recruitment, coordination of assessments, subject retention, data collection and entry, and monitoring compliance, introducing the study, and may obtain consent.

Daylin Anderson is a Staff Research Associate who will conduct clinical assessments and can serve as an interventionist. She may also introduce the study and obtain consent.

Anthony DeBenedetto, MA, is the study Staff Research Associate and the study contact. He will be responsible for subject recruitment, coordination of assessments, subject retention, data collection and entry, and monitoring compliance, and may obtain consent.

Cyrielle Hacher is a Staff Research Associate. She will be responsible for subject recruitment, coordination of assessments, subject retention, data collection and entry, and monitoring compliance, and may obtain consent.

Angela Fish, is Staff Research Associate. She will assist with project coordination and intervention and may obtain consent.

Ana Lopez is the study data manager. He will be responsible for evaluating all data entry accuracy, merging of data files, creation of scales, and evaluating initial frequencies and means. He may also serve as an interventionist and may obtain consent.

Kaylen Moline, MPH is the study recruitment coordinator. She will coordinate all the recruitment and maintenance of the cohort for the study. She may also serve as an interventionist and may obtain consent.

Monica Montoya is a staff research associate and will help with recruitment. She may also serve as an interventionist and may obtain consent.

Jocelyn Schwartz is a Staff Research Associate. She will be responsible for subject recruitment, assessments, subject retention, data collection and entry, and monitoring compliance, and may obtain consent. She may also serve as an interventionist.

Natalie Alamo is a Staff Research Associate who will conduct clinical assessments and can serve as an interventionist. She may also introduce the study and obtain consent.

Georgia Potter is a Staff Research Associate and will conduct clinical assessments and can serve as an interventionist. She may also introduce the study and obtain consent.

Marcia Ellison, Ph.D. is an administrative coordinator and will help with organization of study supplies, conduct clinical assessments, can serve as an interventionist, and can introduce the study and obtain consent.

Maria Linda Burola, MS, is a study recruitment associate. She will assist in the recruitment, screening, and maintenance of the cohort for the study. She may also conduct clinical assessments, serve as an interventionist, and may obtain consent.

Beatriz Gasparetto, BS, is a Staff Research Associate, and will assist in coordination of assessments, subject retention, data collection and entry, and monitoring compliance. She will conduct clinical assessments, may serve as an interventionist, and may obtain consent.

Ellen Pasquale, BS, is a Staff Research Associate, and will assist in coordination of assessments, subject retention, data collection and entry, and monitoring compliance. She will conduct clinical assessments, may serve as an interventionist, and may obtain consent.

Barbara English, BS, is a Staff Research Associate, and she will assist in coordination of assessments, subject retention, data collection and entry, and monitoring compliance. They will conduct clinical assessments, may serve as an interventionist, and may obtain consent.

Renee Laverty, M.Ed, is a Staff Research Associate, and she will assist in coordination of assessments, subject retention, data collection and entry, and monitoring compliance. They will conduct clinical assessments, may serve as an interventionist, and may obtain consent.

Heather Halford, BA, is a Laboratory Assistant, and she will assist in coordination of assessments, subject retention, data collection and entry, and monitoring compliance. They will conduct clinical assessments, may serve as an interventionist, and may obtain consent.

Emily Weed is a Laboratory Assistant and she will assist in coordination of assessments, subject retention, data collection and entry, and monitoring compliance. She will conduct clinical assessments, may serve as an interventionist, and may obtain consent.

Claire Nguyen, Ph.D. is a Staff Research Associate, and she will assist in coordination of assessments, subject retention, data collection and entry, and monitoring compliance. She will conduct clinical assessments, may serve as an interventionist, and may obtain consent.

Graduate students and recent graduate program graduates: The following graduate students and recent program graduates will conduct clinical assessments and can serve as interventionists. They may introduce the study and obtain consent: Zoe Mestre, Michael Manzano and Nicole Virzi, Jessica Willis.

Research assistants: The following undergraduate research assistants will help conduct assessments, introduce the studies to the participants, and may obtain consent. They may also assist with recruitment, group material preparation, data entry, and obtaining heights/weights: Danielle

Gelsinger, Nawal Helo, Jemima Degamo, Cailey Grembowski, Isabella Pascua, Andrew Nguyen, Julianne Trejo, Esther Lee, Sierra Marie, Allison Tegner, Natalia Lodl, Rowan Ustoy, Meena Kian, Karina Barajas, Natalie Le, Weixi Chen, Christy Xie, Carmen Garcia-Sevillano, Paige Awtrey, Carlota Conant, Mariah Price, Lysette Desilva, Millan Kanaya, Siobhan Ang, Cody Spiegel, Gabriela Aldrete, Lindsay Hill, Leila Brasfield, Corinne Blucher, Lauren Hamel.

Volunteers: The following volunteers will help oversee the assessments and introduce the studies to the participants, help with recruitment, screening, assessments, and may obtain consent. They also may help assist with group material preparation and obtaining heights/weights:

22. BIBLIOGRAPHY

1. Jelalian E, Saelens BE. Empirically supported treatments in pediatric psychology: pediatric obesity. *J Pediatr Psychol.* 1999;24(3):223-248.
2. Goldschmidt AB, Stein RI, Saelens BE, Theim KR, Epstein LH, Wilfley DE. Importance of early weight change in a pediatric weight management trial. *Pediatrics.* 2011;128(1):e33-39.
3. Epstein LH, Paluch RA, Roemmich JN, Beecher MD. Family-based obesity treatment, then and now: twenty-five years of pediatric obesity treatment. *Health Psychol.* 2007;26(4):381-391.
4. Epstein LH, Valoski A, Wing RR, McCurley J. Ten-year follow-up of behavioral, family-based treatment for obese children. *Jama.* 1990;264(19):2519-2523.
5. Skelton JA, Beech BM. Attrition in paediatric weight management: a review of the literature and new directions. *Obes Rev.* 2011;12(5):e273-281.
6. Berge JM, Wall M, Loth K, Neumark-Sztainer D. Parenting style as a predictor of adolescent weight and weight-related behaviors. *J Adolesc Health.* 2010;46(4):331-338.
7. Rhee KE, Lumeng JC, Appugliese DP, Kaciroti N, Bradley RH. Parenting styles and overweight status in first grade. *Pediatrics.* 2006;117(6):2047-2054.
8. Musher-Eizenman DR, Holub SC. Children's eating in the absence of hunger: the role of restrictive feeding practices. . In: Flamenbaum R, ed. *Childhood obesity and health research.* : Nova Publishers; 2006:135-156.
9. van der Horst K, Kremers S, Ferreira I, Singh A, Oenema A, Brug J. Perceived parenting style and practices and the consumption of sugar-sweetened beverages by adolescents. *Health Educ Res.* 2006.
10. Stein RI, Epstein LH, Raynor HA, Kilanowski CK, Paluch RA. The influence of parenting change on pediatric weight control. *Obes Res.* 2005;13(10):1749-1755.
11. Golan M, Kaufman V, Shahar DR. Childhood obesity treatment: targeting parents exclusively v. parents and children. *Br J Nutr.* 2006;95(5):1008-1015.
12. Forgatch MS, DeGarmo DS. Parenting through change: an effective prevention program for single mothers. *J Consult Clin Psychol.* 1999;67(5):711-724.
13. Gardner F, Burton J, Klimes I. Randomised controlled trial of a parenting intervention in the voluntary sector for reducing child conduct problems: outcomes and mechanisms of change. *J Child Psychol Psychiatry.* 2006;47(11):1123-1132.
14. Webster-Stratton C. Preventing conduct problems in Head Start children: strengthening parenting competencies. *J Consult Clin Psychol.* 1998;66(5):715-730.
15. Webster-Stratton C, Hammond M. Treating children with early-onset conduct problems: a comparison of child and parent training interventions. *J Consult Clin Psychol.* 1997;65(1):93-109.
16. West F, Sanders MR, Cleghorn GJ, Davies PS. Randomised clinical trial of a family-based lifestyle intervention for childhood obesity involving parents as the exclusive agents of change. *Behav Res Ther.* 2010;48(12):1170-1179.
17. Webster-Stratton C. The Incredible Years. http://www.incredibleyears.com/_content.htm. Accessed Sept 12, 2012, 2006.

18. Patterson GR. *Coersive Family Process.* Eugene, OR: Castalia Press; 1982.
19. Webster-Stratton C. Teaching mothers through videotape modeling to change their children's behavior. *J Pediatr Psychol.* 1982;7(3):279-294.
20. Webster-Stratton C, Hancock L. Training for parents of young children with conduct problems: Content, methods, and therapeutic processes. . In: Schaefer CE, Briesmeister JM, eds. *Handbook of Parent Training*. NY: John Wiley & Sons; 1998:98-152.
21. Eisler RM, Hersen M, Agras WS. Effects of videotape and instructional feedback on nonverbal marital interactions: An analogue study. *Behavior Therapy.* 1973;4:510-558.
22. Webster-Stratton C. Enhancing the effectiveness of self-administered videotape parent training for families with conduct-problem children. *J Abnorm Child Psychol.* 1990;18(5):479-492.
23. Reid MJ, Webster-Stratton C, Beauchaine TP. Parent training in head start: a comparison of program response among African American, Asian American, Caucasian, and Hispanic mothers. *Prev Sci.* 2001;2(4):209-227.
24. Dickstein S, Hayden L, Schiller M, Seifer R, San Antonio W. Providence Family Study. Family Mealtime Interaction coding system. Unpublished coding manual.: Brown University School of Medicine: Bradley Hospital; 1994.
25. Burnham KP, Anderson DR. *Model Selection and Multimodel Inference: A Practical Information-Theoretic Approach.* 2nd ed ed: Springer-Verlag.; 2002.
26. Lanza ST, Flaherty BP, Collins LM. Latent class and latent transition analysis. *Handbook of psychology*. 2003.
27. Lo Y, Mendell NR, Rubin DB. Testing the number of components in a normal mixture. *Biometrika.* 2001;88(3):767-778.
28. Arminger G, Stein P, Wittenberg J. Mixtures of conditional mean-and covariance-structure models. *Psychometrika.* 1999;64(4):475-494.
29. Akaike H. A new look at the statistical model identification. *IEEE transactions on automatic control.* 1974;19(6):716-723.
30. Schwarz G. Estimating the dimension of a model. *The annals of statistics.* 1978;6(2):461-464.
31. Muthén B. Should substance use disorders be considered as categorical or dimensional? *Addiction.* 2006;101(s1):6-16.
32. Bull FC, Maslin TS, Armstrong T. Global physical activity questionnaire (GPAQ): nine country reliability and validity study. *J Phys Act Health.* 2009;6(6):790-804.
33. Barratt W. The Barratt Simplified Measure of Social Status (BSMSS) measuring SES. . Indiana State University; 2006.
34. Jakicic JM, Wing RR, Butler BA, Jeffery RW. The relationship between presence of exercise equipment in the home and physical activity level. *Am J Health Promot.* 1997;11(5):363-365.
35. Sallis JF, Grossman RM, Pinski RB, Patterson TL, Nader PR. The development of scales to measure social support for diet and exercise behaviors. *Prev Med.* 1987;16(6):825-836.
36. Prochaska JJ, Rodgers MW, Sallis JF. Association of parent and peer support with adolescent physical activity. *Res Q Exerc Sport.* 2002;73(2):206-210.
37. Matheny APJ, Wachs TD, Ludwig JL, Phillips K. Bringing Order Out of Chaos: Psychometric Characteristics of the Confusion, Hubbub, and Order Scale. *Journal of Applied Developmental Psychology*. 1995;16:429-444.
38. Margolies P, Weintraub S. The revised 56-item CRPBI as a research instrument: Reliability and factor structure. *Journal of Clinical Psychology.* 1977;33:472-476.
39. Schaefer E. Children's reports of parental behavior: an inventory. *Child Development.* 1965;36:413-424.
40. Conger RD, Ge X. Conflict and cohesion in parent-adolescent relations: Changes in emotional expression from early to mid-adolescence. In: Cox M, Brooks-Gunn J, eds. *Conflict and cohesion in families: Causes and consequences*. Mahwah, NJ: Erlbaum; 1999:185-206.
41. Shumow L, Vendell D, Posner J. Harsh, firm, and permissive parenting in low-income families:

Relations to children's academic achievement and behavioral adjustments. *Journal of Family Issues.* 1998;19:483-507.
42. Arnold DS, O'Leary SG, Wolff LS, Acker MM. The parenting scale: a measure of dysfunctional parenting in discipline situations. *Psychological Assessment.* 1993;5:137-144.
43. Gunnarsdottir T, Njardvik U, Olafsdottir AS, Craighead LW, Bjarnason R. The role of parental motivation in family-based treatment for childhood obesity. *Obesity (Silver Spring).* 2011;19(8):1654-1662.
44. Sallis JF, Pinski RB, Grossman RM, Patterson TL, Nader PR. The development of self-efficacy scales for health-related diet and exercise behaviors. *Health Educ Research.* 1988;3(3):283-292.
45. West F, Sanders MR. The Lifestyle Behavior Checklist: a measure of weight-related problem behavior in obese children. *International Journal of Pediatric Obesity*. 2009;4(4):266-273.
46. Haskett ME, Ahern LS, Ward CS, Allaire JC. Factor structure and validity of the parenting stress index-short form. *J Clin Child Adolesc Psychol.* 2006;35(2):302-312.
47. Loyd BH, Abidin RR. Revision of the Parenting Stress Index. *J Pediatr Psychol.* 1985;10(2):169-177.
48. Birch LL, Fisher JO, Grimm-Thomas K, Markey CN, Sawyer R, Johnson SL. Confirmatory factor analysis of the Child Feeding Questionnaire: a measure of parental attitudes, beliefs and practices about child feeding and obesity proneness. *Appetite.* 2001;36(3):201-210.
49. Wardle J, Guthrie CA, Sanderson S, Rapoport L. Development of the Children's Eating Behaviour Questionnaire. *J Child Psychol Psychiatry*. 2001;42(7):963-970.
50. Tanofsky-Kraff M, Theim KR, Yanovski SZ, et al. Validation of the emotional eating scale adapted for use in children and adolescents (EES-C). *Int J Eat Disord.* 2007;40(3):232-240.
51. Mason AE, Vainik U, Acree M, et al. Improving Assessment of the Spectrum of Reward-Related Eating: The RED-13. *Frontiers in Psychology*. 2017;8.
52. Fairburn CG, Beglin SJ. Assessment of eating disorder psychopathology: interview or self-report questionnaire? *International Journal of Eating Disorders.* 1994;16:363-370.
53. Hendy HM, Williams KE, Camise TS, Eckman N, Hedemann A. The Parent Mealtime Action Scale (PMAS). Development and association with children's diet and weight. *Appetite.* 2009;52(2):328-339.
54. Golan M, Weizman A. Reliability and validity of the Family Eating and Activity Habits Questionnaire. *Eur J Clin Nutr.* 1998;52(10):771-777.
55. Spitzer RL, Williams JB, Kroenke K, et al. Utility of a new procedure for diagnosing mental disorders in primary care. The PRIME-MD 1000 study. *Jama.* 1994;272(22):1749-1756.
56. Radloff LS. The CES-D Scale: A self-report depression scale for research in the general population. *Applied Psychological Measurement.* 1977;1(3):385-401.
57. Achenbach TM. Manual for the Child Behavior Checklist/4-18 and 1991 Profile. Burlington, VT: University of Vermont, Department of Psychiatry; 1991.
58. Varni JW, Seid M, Kurtin PS. PedsQL 4.0: reliability and validity of the Pediatric Quality of Life Inventory version 4.0 generic core scales in healthy and patient populations. *Med Care.* 2001;39(8):800-812.
59. Varni JW, Limbers CA. The pediatric quality of life inventory: measuring pediatric health-related quality of life from the perspective of children and their parents. *Pediatr Clin North Am.* 2009;56(4):843-863.
60. Kirby KN, Maraković NN. Delay-discounting probabilistic rewards: Rates decrease as amounts increase. *Psychonomic bulletin & review.* 1996;3(1):100-104.
61. Hunot C, Fildes A, Croker H, Llewellyn CH, Wardle J, Beeken RJ. Appetitive traits and relationships with BMI in adults: Development of the Adult Eating Behaviour Questionnaire. *Appetite.* 2016;105:356-63.

23. FUNDING SUPPORT FOR THIS STUDY

1R01DK106157-01A1

24. BIOLOGICAL MATERIALS TRANSFER AGREEMENT

N/A

25. INVESTIGATIONAL DRUG FACT SHEET AND IND/IDE HOLDER

N/A

26. IMPACT ON STAFF

N/A

27. CONFLICT OF INTEREST

None of the investigators or any of the study personnel has any financial interest in this study. This includes any financial relationships, service on advisory boards, company officer and stock option ownership. All investigators will complete a 700U form on file with Grants and Contracts.

28. SUPPLEMENTAL INSTRUCTIONS FOR CANCER-RELATED STUDIES

N/A

29. OTHER APPROVALS/REGULATED MATERIALS

N/A

30. PROCEDURES FOR SURROGATE CONSENT AND/OR DECISIONAL CAPACITY ASSESSMENT

N/A

Version date: May 11, 2011
