# Supplementary material for: Parenting Training Plus Behavioral Treatment for Children With Obesity: A Randomized Clinical Trial
Source: JAMA Netw Open. 2025 May 5;8(5):e258398. doi: 10.1001/jamanetworkopen.2025.8398 (PMC12053569; doi:10.1001/jamanetworkopen.2025.8398)
Supplement: Supplement 2. — eTable 1. Timeline of Treatment Cohorts eTable 2. Content of FBT and FBT Plus PT Treatment Sessions eTable 3. Child Weight Status by Group at Baseline, Posttreatment, and 6- and 12-Month Follow-up eFigure. Probability of Attrition in FBT vs FBT Plus PT During the 6-Month Intervention [file jamanetwopen-e258398-s002.pdf]

Supplementary Online Content

Rhee KR, Corbett T, Patel S, et al. Parenting training plus behavioral treatment for children with obesity: a randomized clinical trial. *JAMA Netw Open*. 2025;8(5):e258398. doi:10.1001/jamanetworkopen.2025.8398

eTable 1. Timeline of Treatment Cohorts

eTable 2. Content of FBT and FBT Plus PT Treatment Sessions

eTable 3. Child Weight Status by Group at Baseline, Posttreatment, and 6- and 12-Month Follow-up

eFigure. Probability of Attrition in FBT vs FBT Plus PT During the 6-Month Intervention

This supplementary material has been provided by the authors to give readers additional information about their work.

© 2025 Rhee KR et al. *JAMA Network Open*.

eTable 1: Timeline of treatment cohorts

	Treatment Start Date	Treatment End Date	6-month follow-up	12-month follow-up
**Cohort 1**	March 2018	August 2018	February – April 2019	August – November 2019
**Cohort 2**	October 2018	March 2019	September – November 2019	March – June 2020
**Cohort 3**	June 2019	November 2019	May – July 2020	October 2020 – January 2021
**Cohort 4**	November 2019	April 2020	October – December 2020	March – June 2021
**Cohort 5**	March 2020	August 2020	February – April 2021	July - October 2021
**Cohort 6**	January 2021	June 2021	December 2021 – February 2022	July 2022 – November 2022

The first three cohorts received all of their treatment in-person. A COVID-19 stay-at-home order was issued in California on March 19, 2020. Cohort 4 received their first 16 sessions in-person and was switched to remote (via Zoom©) for the last 4 sessions. Cohorts 5 and 6 received all of their treatment remotely. Assessments occurred at baseline, during treatment, post-treatment (month 6), 6-month follow-up (month 12), and 12-month follow-up (month 18). During the COVID-19 pandemic, participants were provided with blue tooth-enabled scales (Withings©) to collect weight information and tape measures to collect height.

eTable 2: Content of FBT and FBT+PT treatment sessions

Session #	FBT	FBT + PT	Active parenting skills training elements added to FBT + PT
1	Introduction: Energy balance, self-monitoring, family meetings	Introduction: Energy balance, self-monitoring, family meetings	Emphasize the importance of parent attention and special time
2	Healthy Eating	Healthy Eating	Emphasize persistence coaching when eating healthy
3	Stimulus Control/ Home Environment	Positive Parenting	Positive reinforcement, reward chart, modeling healthy behavior, effective use of praise and encouragement
4	Physical Activity	Stimulus Control/ Home Environment	Avoid pairing praise and criticism, look for the positive, avoid sarcasm
5	Motivation Systems/ Positive Parenting	Physical Activity	Teach through encouragement - promote pro-social behaviors with contingent positive reinforcement
6	Behavior Chains	Behavior Chains	Teach through encouragement - turn complaints into positive recommendations, make positive requests
7	Problem-Solving	Setting Limits	Encouraging cooperation - provide children with clear, firm, and respectful directions to increase compliance
8	Healthy Lifestyle/ Sedentary Behaviors	Problem-Solving	Problem-solving with active skills learning, promoting process not solution
9	Problem-Solving: High-Risk Situations	Healthy Lifestyle/ Sedentary Behaviors	Setting clear limits, using non-coercive discipline strategies, and follow-through with limits
10	Motivation	Problem-Solving: High-Risk Situations	Ignoring misbehavior, natural consequences, time-out consequences
11	Responsibility	Motivation	Staying motivated: pros and cons to making healthy choices,<br>setting up routines and clear expectations
12	Review: Open forum to address challenges	Responsibility	Monitoring children’s activities when they are away from home
13	Behavior Chains: High-Risk Situation Activities	Tricky Hunger/ Emotional Eating	Observing and managing your emotions
14	Tricky Hunger/ Emotional Eating	Body Image/ Teasing	How to give and get support from family members, positive self-talk
15	Shopping on a Budget	Social Support/ Sabotage	Managing conflicts with other family members
16	Meal Planning	Shopping on a Budget	Review of setting limits
17	Body Image/ Teasing	Meal Planning	Review of positive parenting styles: Importance of parent attention and special time
18	Social Support/ Sabotage	Review: Open forum to address challenges	Persistence coaching
19	Relapse Prevention	Relapse prevention	Dealing with child discouragement, build on their strengths, motivate with praise
20	Graduation: Review	Graduation: Review	Review curriculum: emphasize parent autonomy to implement behavioral changes & positive parenting techniques

FBT = Family-based Behavioral Treatment
FBT+PT = Family-based Behavioral Treatment + Parenting Training

© 2025 Rhee KR et al. *JAMA Network Open*.

eTable 3: Child weight status by group at baseline, post-treatment (6 months), and 6- and 12-month follow-up

	FBT treatment group				FBT + PT treatment group			
	Baseline	Post-treatment	6-month Follow-up	12-month Follow-up	Baseline	Post-treatment	6-month Follow-up	12-month Follow-up
BMI percentile	97.41 (2.33)	93.93 (5.04)	94.32 (4.61)	92.72 (10.40)	97.22 (3.64)	93.32 (5.27)	93.64 (5.43)	93.34 (6.21)
BMI z-score	2.25 (0.77)	1.68 (0.43)	1.72 (0.45)	1.66 (0.55)	2.31 (0.85)	1.64 (0.46)	1.68 (0.49)	1.67 (0.51)
%BMIp95	121.54 (22.75)	103.83 (13.17)	105.15 (14.20)	104.52 (15.10)	122.92 (24.34)	102.90 (14.41)	104.14 (15.29)	104.11 (15.71)

Weight status is presented for both groups as estimated means (SD) at baseline, post-treatment (month 6), and 6- and 12-month follow-up (months 12 and 18).

© 2025 Rhee KR et al. *JAMA Network Open*.

eFigure: Probability of attrition in FBT vs. FBT+PT during the 6-month intervention

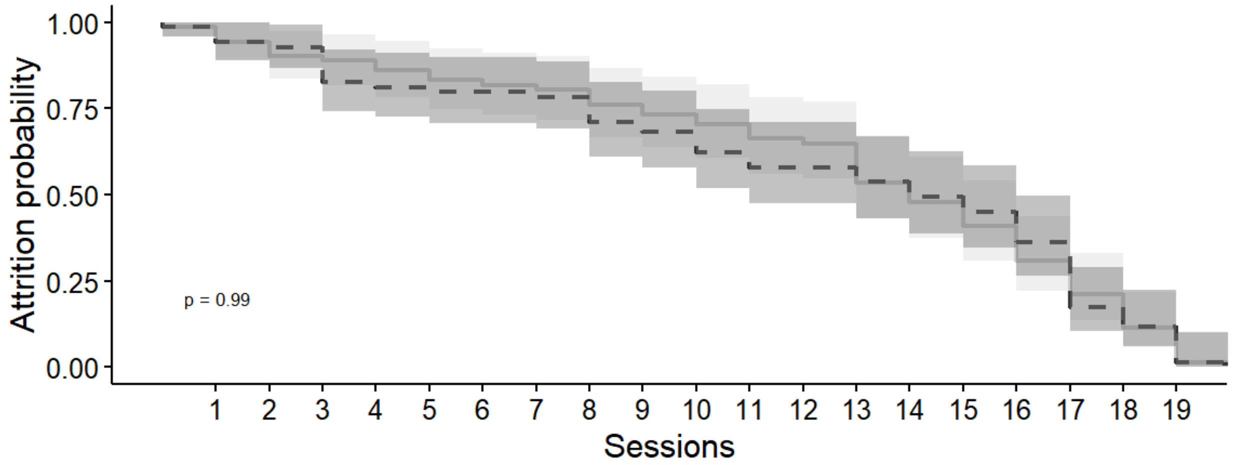

Number of parent-child dyads who attended treatment

FBT	71	70	67	64	63	61	59	58	57	54	52	50	47	46	38	34	29	22	15	8	[illegible]
FBT + PT	69	68	65	64	57	56	55	55	54	49	47	43	40	40	37	34	31	25	12	8	[illegible]

Treatment: FBT FBT + PT

A survival plot was created to demonstrate the probability of attrition during treatment based on the number of treatment sessions families attended. We included covariates to account for potential variation in outcome that may be related to differential drop-out between demographic groups. There was no difference in the probability of attrition between FBT and FBT+PT (HR=1.01, 95%CI, 0.72-1.43, p=0.95). Furthermore, there were no significant associations between sex (HR=1.29, 95%CI, 0.88-1.89, p=0.19), age (HR=1.00, 95%CI 0.99-1.01, p=0.84), or racial/ethnic groups (non-Hispanic White vs. Hispanic (HR=0.80, 95%CI 0.50-1.29, p=0.36), multiple races vs. Hispanic (HR=0.99, 95%CI, 0.68-1.45, p=0.96)) and risk of drop-out.

© 2025 Rhee KR et al. *JAMA Network Open*.
