# Supplementary material for: Parenting Training Plus Behavioral Treatment for Children With Obesity: A Randomized Clinical Trial
Source: JAMA Netw Open. 2025 May 5;8(5):e258398. doi: 10.1001/jamanetworkopen.2025.8398 (PMC12053569; doi:10.1001/jamanetworkopen.2025.8398)
Supplement: Supplement 3. — Data Sharing Statement [file jamanetwopen-e258398-s003.pdf]

Data Sharing Statement

Rhee. Parenting Training Plus Behavioral Treatment for Children With Obesity. *JAMA Netw Open*. Published May 05, 2025. doi:10.1001/jamanetworkopen.2025.8398

Data
Additional Information: http://clinicaltrials.gov. Identifier: NCT02976636
Data available: Yes
Data types: Deidentified participant data, Data dictionary
How to access data: k1rhee@health.ucsd.edu
When available: beginning date: 01-31-2026

Supporting Documents
Document types: None

Additional Information
Who can access the data: Deidentified individual-level participant data and code book will be made available one year after publication to researchers who provide a methodologically sound proposal. Proposals should be submitted to Dr. Kay Rhee at k1rhee@health.ucsd.edu for review and approval. Data use agreements will be required.
Types of analyses: For any purpose
Mechanisms of data availability: after approval of a proposal and with a signed data use agreement.
